# Time‑resolved transcriptome analysis during transitions of sulfur nutritional status provides insight into triacylglycerol (TAG) and astaxanthin accumulation in the green alga *Chromochloris zofingiensis*

**DOI:** 10.1186/s13068-020-01768-y

**Published:** 2020-07-17

**Authors:** Xuemei Mao, Yongmin Lao, Han Sun, Xiaojie Li, Jianfeng Yu, Feng Chen

**Affiliations:** 1 Shenzhen Key Laboratory of Marine Microbiome Engineering, Institute for Advanced Study, Shenzhen University, Shenzhen, 518060 China; 2 College of Physics and Optoelectronic Engineering, Shenzhen University, Shenzhen, 518060 China; 3 Institute for Innovative Development of Food Industry, Shenzhen University, Shenzhen, 518060 China

**Keywords:** *Chromochloris zofingiensis*, Transcriptomic dynamics, Lipid metabolism, Astaxanthin, Sulfur-starvation

## Abstract

**Background:**

*Chromochloris zofingiensis*, an oleaginous microalga, is a promising feedstock for the co-production of triacylglycerol (TAG)-based biodiesel and the high-value product astaxanthin. To reveal the molecular mechanism of TAG and astaxanthin biosynthesis during transitions of sulfur nutritional status, namely sulfur-starvation (SS) and sulfur-replenishment (SR), the physiological responses and the transcriptomic dynamics of *C. zofingiensis* were examined.

**Results:**

The results revealed a reversible TAG and astaxanthin accumulation under SS, which is correlated with the reduction of cell growth and protein content, indicating the reallocation of carbon. By correlating the data on the physiological and transcriptional responses to different sulfur nutritional status, a model for the underlying mechanism of TAG and astaxanthin accumulation in *C. zofingiensis* was postulated, which involved up-regulation of key genes including diacylglycerol acyltransferase (*DGTT5*) and beta-carotene ketolase (*BKT1*), increased energy and NADPH supply by elevating the tricarboxylic acid (TCA) cycle and the oxidative pentose phosphate (OPP) pathway, and the increased carbon precursors (pyruvate and acetyl-CoA) through central carbon metabolism. In addition, the net enhancement of the de novo biosynthesis of fatty acids and the re-direction of the terpenoid precursors toward the branch catalyzed by lycopene beta cyclase (*LCYb*) and *BKT1* escalated the substrate availability for the biosynthesis of TAG and astaxanthin, respectively.

**Conclusions:**

In this study, the time-resolved transcriptional analysis of *C. zofingiensis* under SS and SR conditions was reported for the first time to elucidate the regulatory roles of key enzymes, including *DGTT5*, *BKT1* and *LCYb*, in the underlying mechanisms of TAG and astaxanthin accumulation.

## Background

Microalgae are promising candidates for production of the third-generation biodiesel; owing to advantages of high lipid content, fast growth rate and the capacity for photoautotrophic growth using CO_2_ as carbon source [1, 2]. Meanwhile, microalgae have also been considered as hosts for production of high-value products such as carotenoids, polyunsaturated fatty acids and polysaccharides, which are widely applied in the cosmetic, nutraceutical and pharmaceutical industries [3, 4]. Recently, an integrative microalgae biorefinery approach was proposed to promote the sustainability and the net commercial benefit via co-production of various products including high-value products and biofuel [5, 6]. *Chromochloris zofingiensis* is able to accumulate TAG and astaxanthin under stress conditions, such as nutrient depletion and high light [7, 8]. However, knowledge on the metabolism of lipids and astaxanthin in *C. zofingiensis* is still sparse; especially the mechanisms and factors involved in the transcriptional regulation is largely unclear, despite key genes associated with the TAG and astaxanthin biosynthesis pathways having been identified via bioinformatic analysis [9–11]. It is important to determine the parameters on the stress-induced accumulation of TAG and astaxanthin in *C. zofingiensis* and study the underlying metabolic mechanisms, the knowledge from which would aid further development of the co-production of microalgal biodiesel and high-value products.

Microalgae respond rapidly to environmental stresses such as nutrient depletion, salt stress and intense illumination [12, 13]. One of the best characterized stress factors is nitrogen deprivation which is known to induce lipid or/and carotenoids accumulation in a wide variety of microalgae [12, 14, 15]. Recently, several reports have described the underlying molecular mechanism for TAG or astaxanthin accumulation in *C. zofingiensis* under nitrogen starvation or glucose feeding [10, 16, 17]. While the impacts of the C/N ratio or nitrogen starvation have drawn much attention, little is known about that of the C/S ratio or sulfur-starvation (SS). Microalgae and higher plants require sulfur to produce numerous essential metabolites for cell growth and reproduction, such as sulfur-containing amino acids cysteine and methionine. Studies on sulfur acquisition and metabolism in higher plants have yielded fundamental understanding of the regulatory processes for sulfur assimilation, but with less focus on the regulatory mechanism of lipids or carotenoids biosynthesis [18, 19]. As for microalgae, previous study indicated that SS could be a more efficient approach to induce TAG production than N starvation, because prolonged cultivation under SS resulted in higher TAG accumulation in *Chlamydomonas reinhardtii* [20]. The induction of lipid biosynthesis was in tandem with a decrease of starch content in the green microalga *Parachlorella kessleri* [21]. In addition, SS induced the accumulation of astaxanthin in *Haematococcus pluvialis*, an important carotenoid with high market value [22]. Our previous research has revealed the simultaneous induction of TAG and astaxanthin accumulation under SS condition in *C. zofingiensis*, in which the yield of TAG and astaxanthin was even higher than that of N and P starvation [7].

The transcriptional regulatory mechanisms involved in TAG and astaxanthin accumulation under SS are unclear, and only a few regulators have been identified relating to lipid accumulation during SS [23, 24]. Therefore, analysis on the dynamics of the transcriptome of cells during sulfur-starvation would elucidate the underlying mechanism and shed light on the identification of gene targets. Furthermore, the transcriptomic analysis method is also applicable to study the physiological change through the recovery period of sulfur deprivation which has yet been reported. Previous study on the transcriptional responses to sulfur assimilation or TAG accumulation under SS condition was based on the model microalga *C. reinhardtii* [24, 25]. These studies have revealed a redirection of metabolic carbon flow from protein toward TAG synthesis in S-starved cells. Two kinases in *C. reinhardtii*, SNRK2.2 and TAR1, were indicated as TAG accumulation regulators responsible for the cellular responses to SS [23, 24]. However, due to the low abundance of biodiesel precursors and high-value compounds, *C. reinhardtii* is not ideal for productivity analysis. *C. zofingiensis* was recently proposed as a new model microalga to study the co-production of TAG and astaxanthin, due to its ability to accumulate TAG and astaxanthin, and robust growth characteristics under heterotrophic, phototrophic and mixotrophic cultivation conditions [10, 17]. Moreover, the whole genome of *C. zofingiensis* has been sequenced and the respective genetic engineering tools have been developed [9, 26]. Thus, *C. zofingiensis* was used in this study to investigate the dynamic metabolic responses to the transitions of sulfur status.

To reveal the responses of *C. zofingiensis* under SS and sulfur-replenishment (SR), physiological parameters referring to cell growth, photosynthetic activity, lipid content and carotenoid profiles were examined during the two-phase phototrophic cultivation. The time-resolved transcriptome was analyzed to elucidate underlying mechanisms of TAG and astaxanthin accumulation during sulfur-starvation, and identify potential target genes for metabolic engineering to further improve the capacity for TAG and astaxanthin production.

## Results

### Sulfur-starvation leads to cell growth inhibition and cellular ROS accumulation

**Fig. 1:**
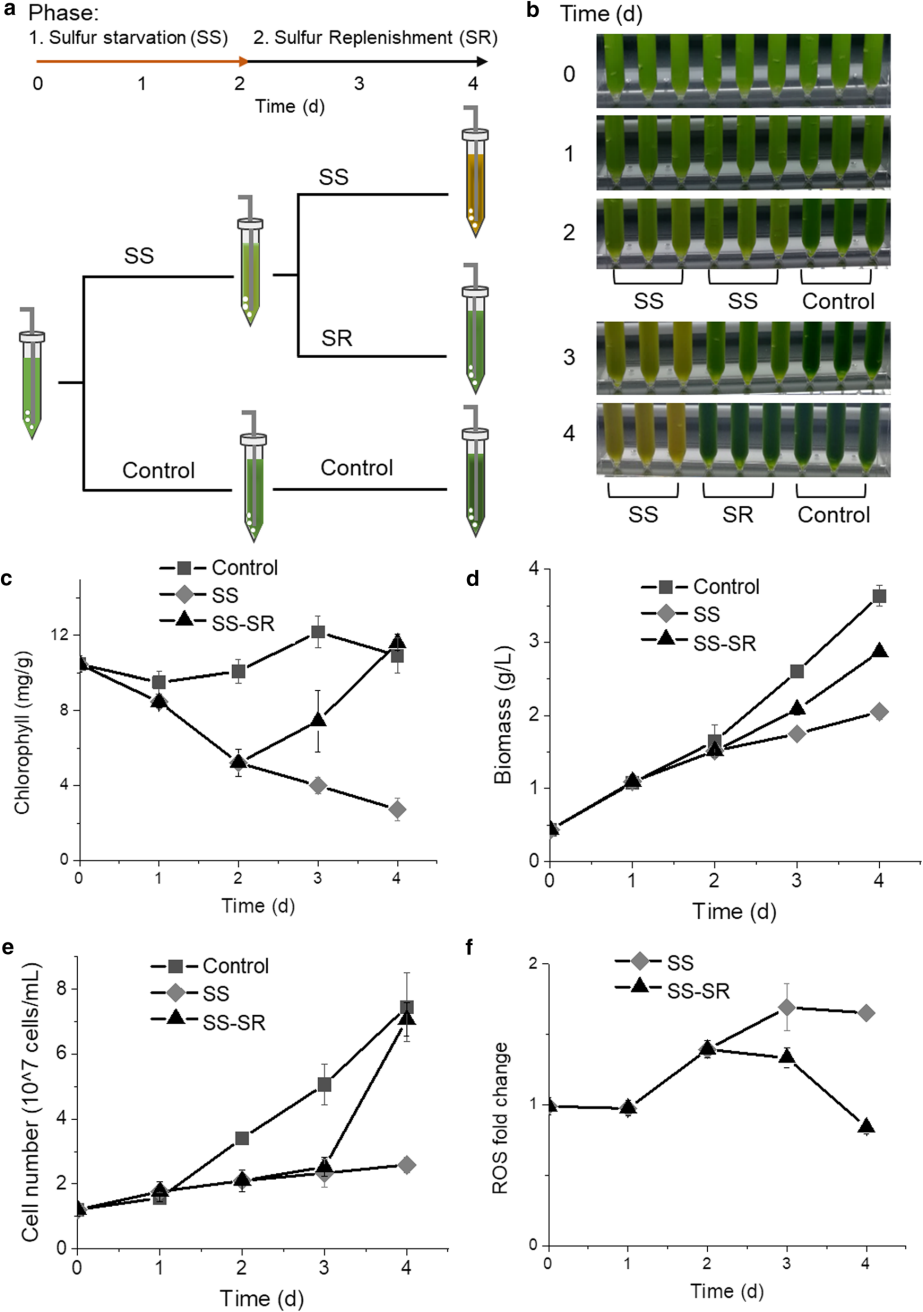
Growth of *C. zofingiensis* under SS and SR. **a** Schematic representation of the experiment design. **b***C. zofingiensis* culture status. **c** Chlorophyll content. **d** Biomass dynamics. **e** Cell number change. **f** ROS level changes with control group as standard. SR stage in SS–SR group was started after 2 days. DW, dry weight

A two-phase cultivation was conducted to assess the effects of SS and SR (Fig. 1a). In Phase I, *C. zofingiensis* cells cultivated in Kuhl medium were transferred into SS medium for sulfur-starvation treatment and the positive control cells were transferred into fresh Kuhl medium, then both cultures were incubated for a 2-day period. Cultures in the SS group turned light green after Phase I (Fig. 1b), and the chlorophyll content was substantially decreased (Fig. 1c). Then cells were transferred to Phase II and 1 mM Na_2_SO_4_ was added into the cultures (SR group), while the respective control groups, including the SS and the positive control groups, were kept unchanged. After resupplementation of sulfate, the yellow SS cultures reverted to green due to the recovery of chlorophyll content (Fig. 1c). The increase of biomass was repressed under SS along with a reduced cell proliferation rate; and the suppression on biomass and cell number was lifted after SR (Fig. 1d, e). The cellular level of ROS (reactive oxygen species), a group of signaling molecules regulating various physiological responses, was examined during this reversible process. The cellular level of ROS in SS cells was significantly higher (*p* < 0.05), and reached 1.39-fold and 1.65-fold of the control group at the end of Phase I and II, respectively (Fig. 1f). After SR, the ROS level gradually decreased to the same level as the control group, which is consistent with previous report that the ROS production was associated with the environmental stresses such as nitrogen depletion or intense illumination [27, 28].

### Under sulfur-starvation, TAG and astaxanthin accumulate while protein decreases

**Fig. 2:**
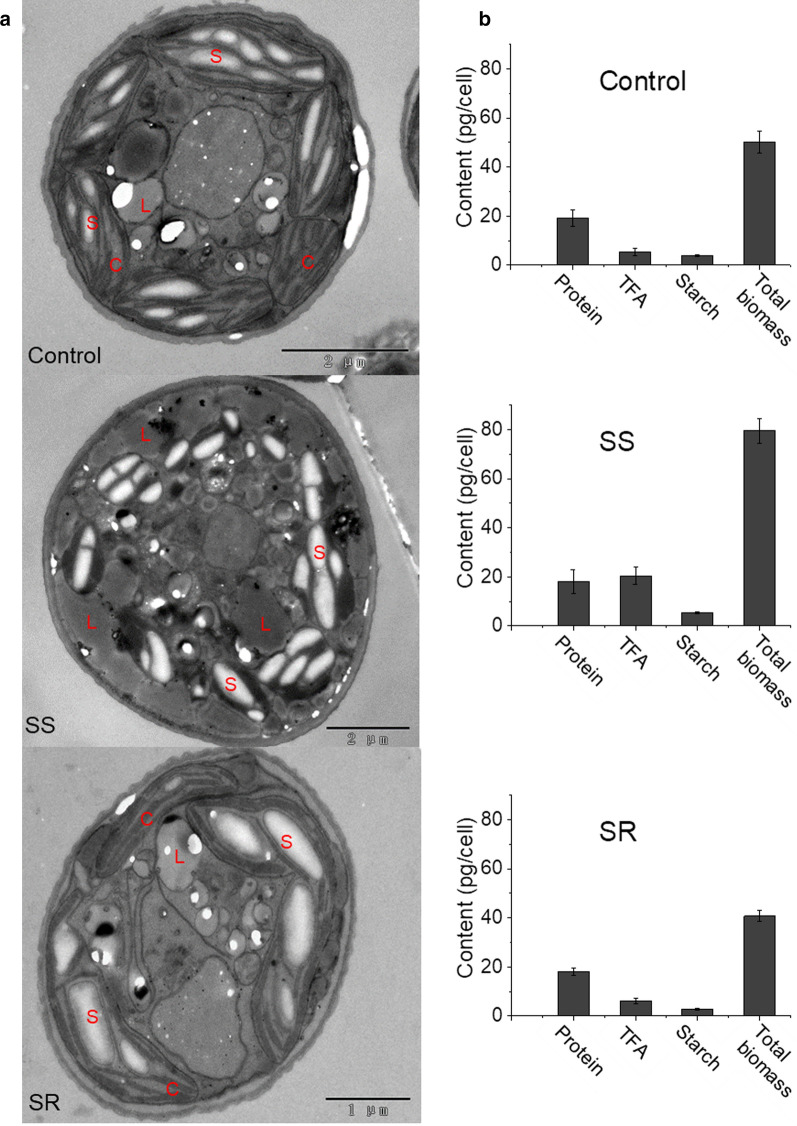
Subcellular structures (**a**) and (**b**) biochemical changes after 4 days. L, lipid droplet; S, starch granule; C, chloroplast

After the 4-day sulfur-starvation–replenishment experiments, the structural integrity and the biochemical composition of the biomass from each group were examined via 2D transmission electron microscopy (TEM) (Fig. 2a). The structure of the thylakoid membranes in the chloroplast was severely damaged under the SS condition, but restored after SR. Meanwhile, the amount of lipid droplets in the SS samples was substantially more than that of the positive control and the SR groups. The abundance of protein, starch, and total fatty acids (TFA) in each sample was also analyzed (Fig. 2b). The cell weight and cell size were increased in SS cultures. Among the three major components, protein accounted for approximately 40% of biomass in the positive control and SR cells on Day-4. It was shown that in the SS group, the protein content was decreased to 22.4% of biomass dry weight, although the protein level per cell was similar to control cells; in contrast, the TFA content reached 25.5% of the dry weight (compared with 10.6% in the positive control group), and the TFA level per cell was increased threefold, which is a significant (*p* < 0.05) stoichiometric change to the cellular composition. The variation in the TFA content between the SR and the positive control groups is insignificant (*p* > 0.05). Although the starch level per cell was increased in SS cells, the percentage of starch in biomass unchanged among all groups.

**Fig. 3:**
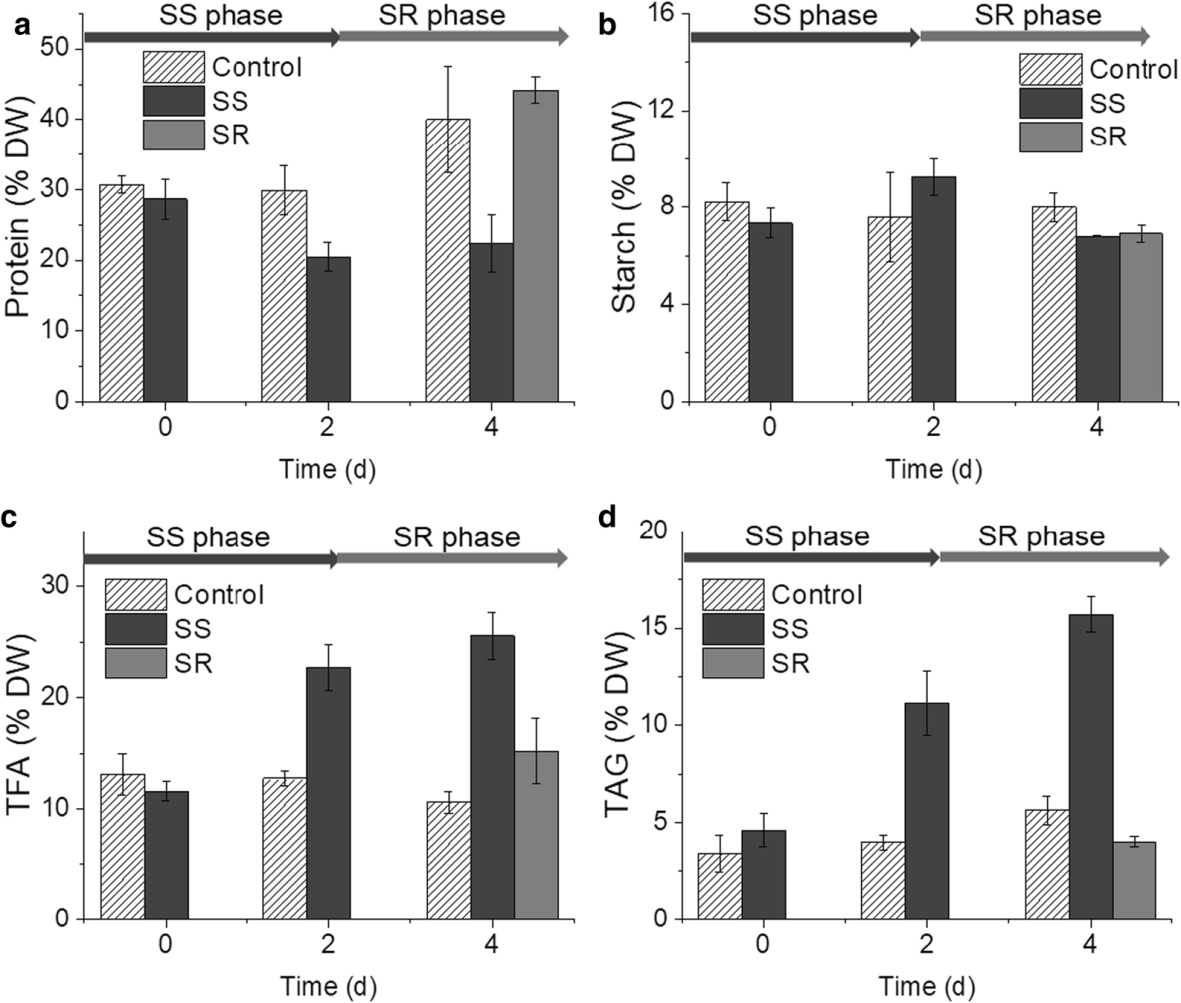
Time course of protein (**a**), starch (**b**), TFA (**c**) and TAG (**d**) content. DW, dry weight

The time courses of protein, starch, TFA and TAG content were also plotted to show the dynamics of the cellular composition (Fig. 3). Under SS condition, the protein content was stably kept at the lowest level from Day-2 to Day-4, whereas that of the SR samples restored to the pre-starvation level in 2 days (Fig. 3a). The starch level remained unchanged in all groups under all tested conditions (Fig. 3b). The TFA level reached maxima under SS condition, which was 22.7% of the total dry weight on Day-2 and 25.5% on Day-4, and substantially decreased after replenishment of sulfate (Fig. 3c). The TAG content showed a similar pattern to that of the TFA, which peaked at 15.7% of the total biomass on Day-4 under the SS condition (Fig. 3d). Therefore, the TFA accumulation in *C. zofingiensis* is correlated with the reduction of the protein content.

**Fig. 4:**
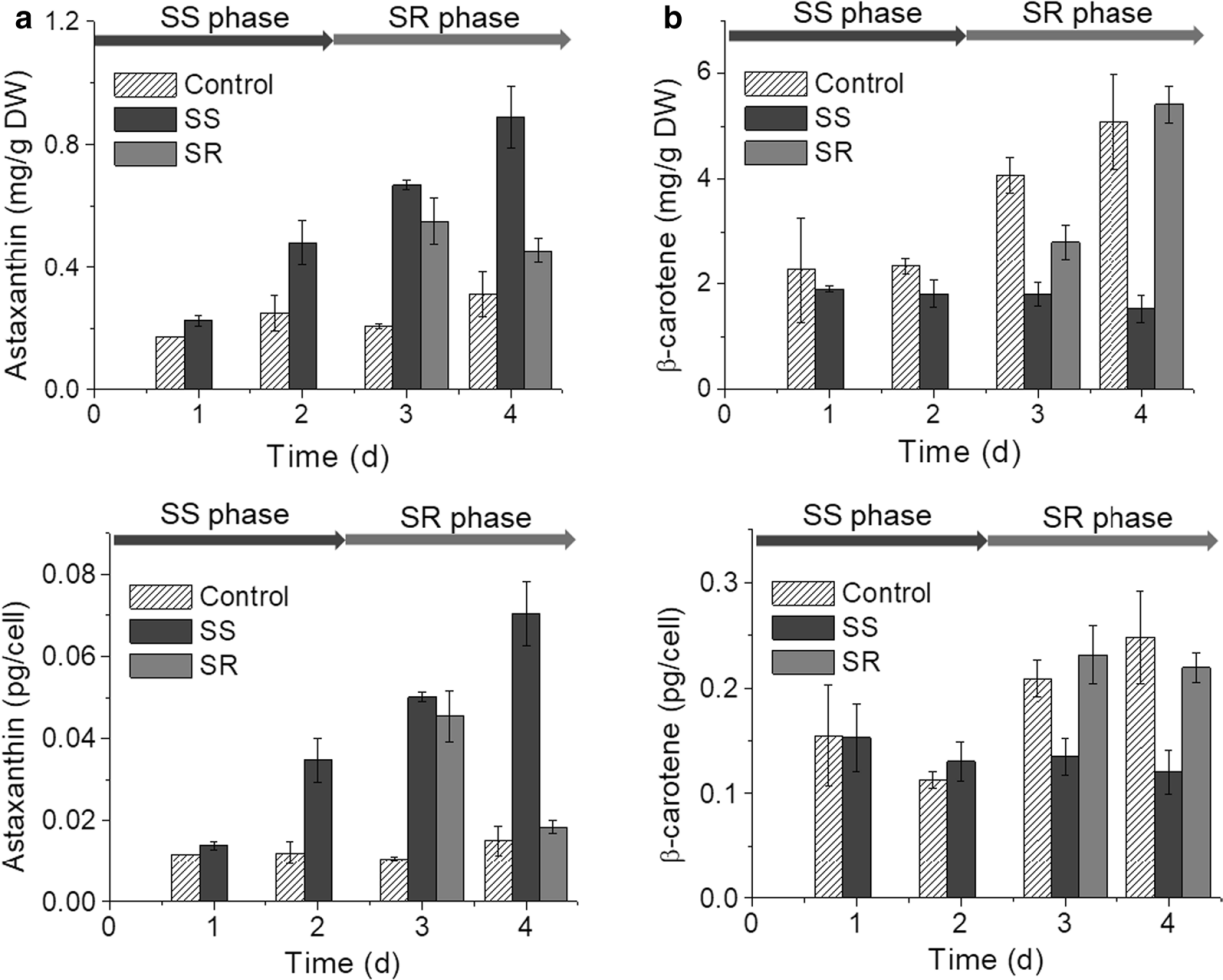
Time course of astaxanthin (**a**) and β-carotene (**b**) content. DW, dry weight

The content of astaxanthin, a high-value compound, was significantly increased in *C. zofingiensis* under the SS condition, and the level per cell was also increased (Fig. 4a). The majority of astaxanthin was esterified with fatty acids as mono-ester and di-esters (Additional file 4: Fig. S1a). In contrast, the level of β-carotene, the precursor of astaxanthin, was significantly lower under the SS condition (*p* < 0.05) (Fig. 4b). Interestingly, these responses were also reversible after sulfate-replenishment as the contents of both astaxanthin and β-carotene were gradually recovered to a similar level as the control group. In addition, the contents of α-carotene in SS cells were lower than control and SR cells (Additional file 4: Fig. S1b), and the content of total carotenoids was decreased under SS (Additional file 4: Fig. S1c).

### Transcriptome analysis reveals the metabolic adjustments during SS and SR

**Fig. 5:**
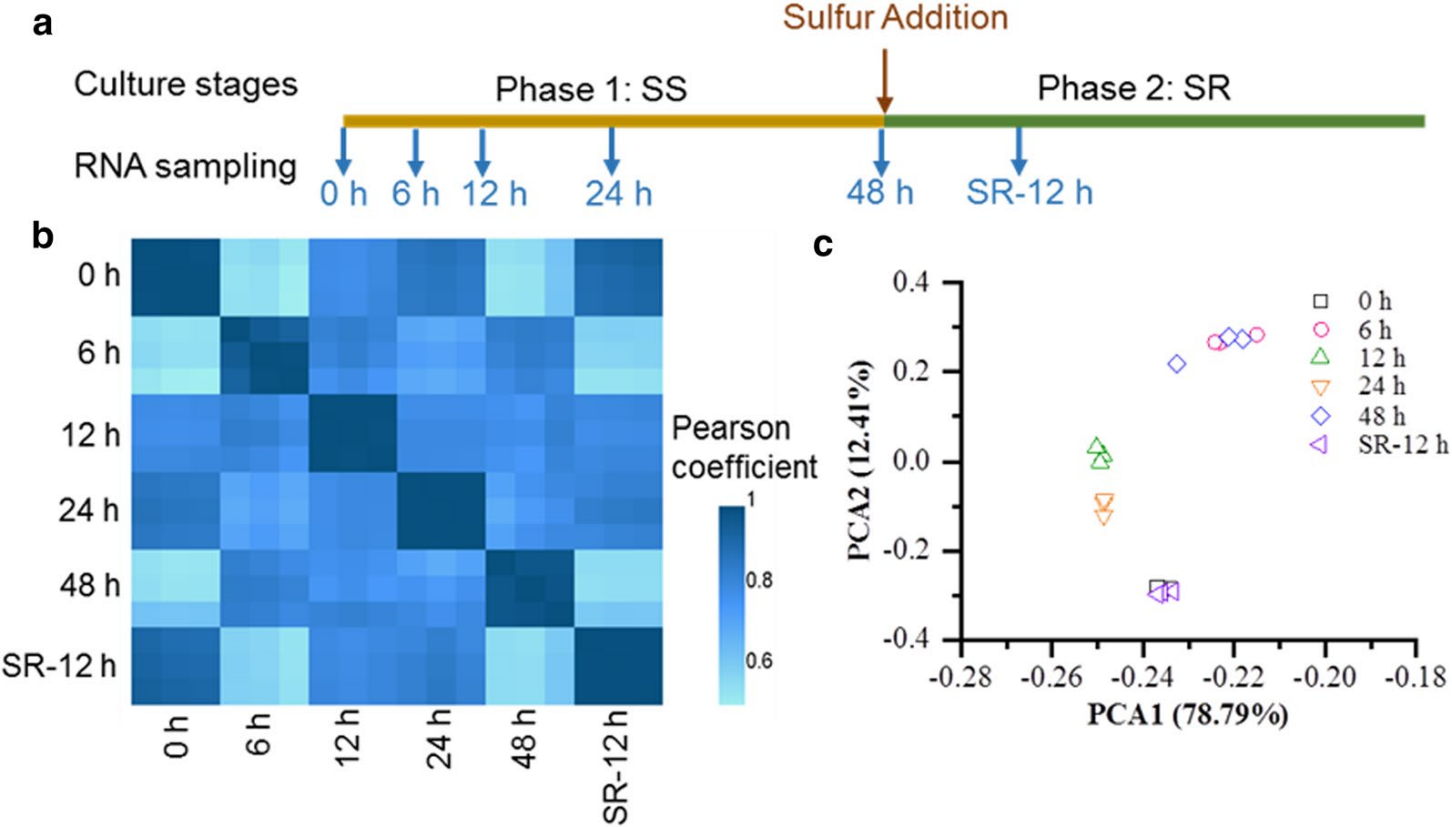
Overview of the transcriptome analysis. **a** Experimental design of RNA sampling during transitions of sulfur nutritional status. **b** Pearson correlation analysis of all gene expression levels. **c** Principal component analysis (PCA) analysis

To investigate the transcriptional regulations associated with sulfur-mediated metabolic responses, total RNA was extracted from the respective cells at the following time points: 0 h, 6 h, 12 h, 24 h and 48 h through the SS phase, and 12 h into the SR phase (referred as SR-12 h), 3 biological replicates were taken at each time point (Fig. 5a). For each sample, approx. 24 million clean reads were obtained and mapped into the reference genome. In total, 15,224 genes were detected (See Additional file 1). According to the Pearson correlation coefficient and the PCA analysis, the transcription profiles of the 18 samples generated in this study had high reproducibility among the three biological replicates (Fig. 5b, c). Moreover, the total expression level of the samples taken at 6 h and 48 h showed greatest difference compared with the control group (0 h), while that from the samples taken at 12 h and 24 h were less obvious. The expression level of the SR-12 h samples was most similar to that of 0 h; together, the changes in the transcriptome pattern correlated well with the changes of the physiological and biochemical parameters through each phase.

The changes in transcript abundance relative to the control group (0 h) were expressed as log2FoldChange (FC), and the genes with log2FC ≥ 1 or ≤ − 1 and *p*_adj_ ≤ 0.05 were regarded as differentially expressed genes (DEGs). In total, 9101 genes were recognized as DEGs from all groups. As shown in the volcano plots (Additional file 4: Fig. S2), the 6 h group contains more DEGs, with 2189 up-regulated genes and 2953 down-regulated genes, followed by the 12 h group with 1349 up-regulated genes and 2079 down-regulated genes. Thus, a global transcriptional cascade was stimulated by the SS stress within 6 h, and the reduction of the transcripts level during the 6- to s12-h period indicated cells begin to acclimate to the SS condition, and the transcriptional responses were effectively arrested by sulfur-resupplementation.

To elucidate the key genes associated with the cellular responses to sulfur stress and recovery, DEGs of 6 h (compared with 0 h) and SR-12 h (compared with 48 h) were analyzed using the reference database “KEGG” for pathway classification and functional enrichment (Additional file 4: Fig. S3). The KEGG category containing most DEGs from the 6 h sample was “Global and overview maps”, then followed by “Translation”, “Folding, sorting and degradation”, “Carbohydrate metabolism”, “Amino acid metabolism” and “Lipid metabolism” (Additional file 4: Fig. S3a). That of the DEGs from the SR-12 h sample are “Translation”, “Carbohydrate metabolism” and “Lipid metabolism”, which indicated that SS stress and SR reversion mainly act upon protein synthesis, carbohydrate and lipid metabolism (Additional file 4: Fig. S3b). The KEGG pathways significantly enriched (*p* < 0.1) were listed (see Additional file 2). There are 8 pathways under the “Global and overview maps” directory, among which “fatty acid metabolism” and “biosynthesis of secondary metabolites” were enriched in DEGs of the SS 6 h sample. For identification of key functional elements, 20 most enriched KEGG categories were showed (Additional file 4: Fig. S3c, d). DEGs of the 6 h sample were highly enriched in “Photosynthesis-antenna proteins” and “Proteasome”. The DEGs in the “Photosynthesis-antenna proteins” category were substantially down-regulated and that of the “Proteasome” category were up-regulated (Additional file 4: Fig. S4). In tandem with previously observed protein content reduction and thylakoid damage (Fig. 2), the transcriptome analysis provided additional evidence on SS-mediated photosynthesis system and protein degradation. DEGs in significantly enriched KEGG pathways (*p* < 0.1) were listed (Additional file 2), and those appeared in both SS 6 h and SR 12 h were selected for hierarchical cluster analysis to sort these genes according to the similarity between their expression profiles and elucidate the networks of co-expressed genes (Additional file 4: Fig. S5). Not surprisingly, DEGs in “Photosynthesis-antenna proteins” and “Porphyrin and chlorophyll metabolism” categories clustered together sharing similar transcriptional patterns that down-regulated under SS and reverted after SR. Gene ontology (GO) analysis was also conducted and the distribution of most enriched GO terms for biological processes, molecular functions, and cellular components was listed (See Additional file 2). In SS 6 h group, GO terms associated with ribosome were highly enriched, and GO terms linked to thylakoid, chloroplast and photosynthesis were highly enriched in SR 12 h group. The top 100 genes having highest differential expression in SS 6 h group (50 for up-regulation and 50 for down-regulation) were identified (See Additional file 3), and most of them were unknown in function according to sequence BLAST in Non-Redundant Protein Sequence Database.

### The transcriptional impact of SS on TAG biosynthesis

**Fig. 6:**
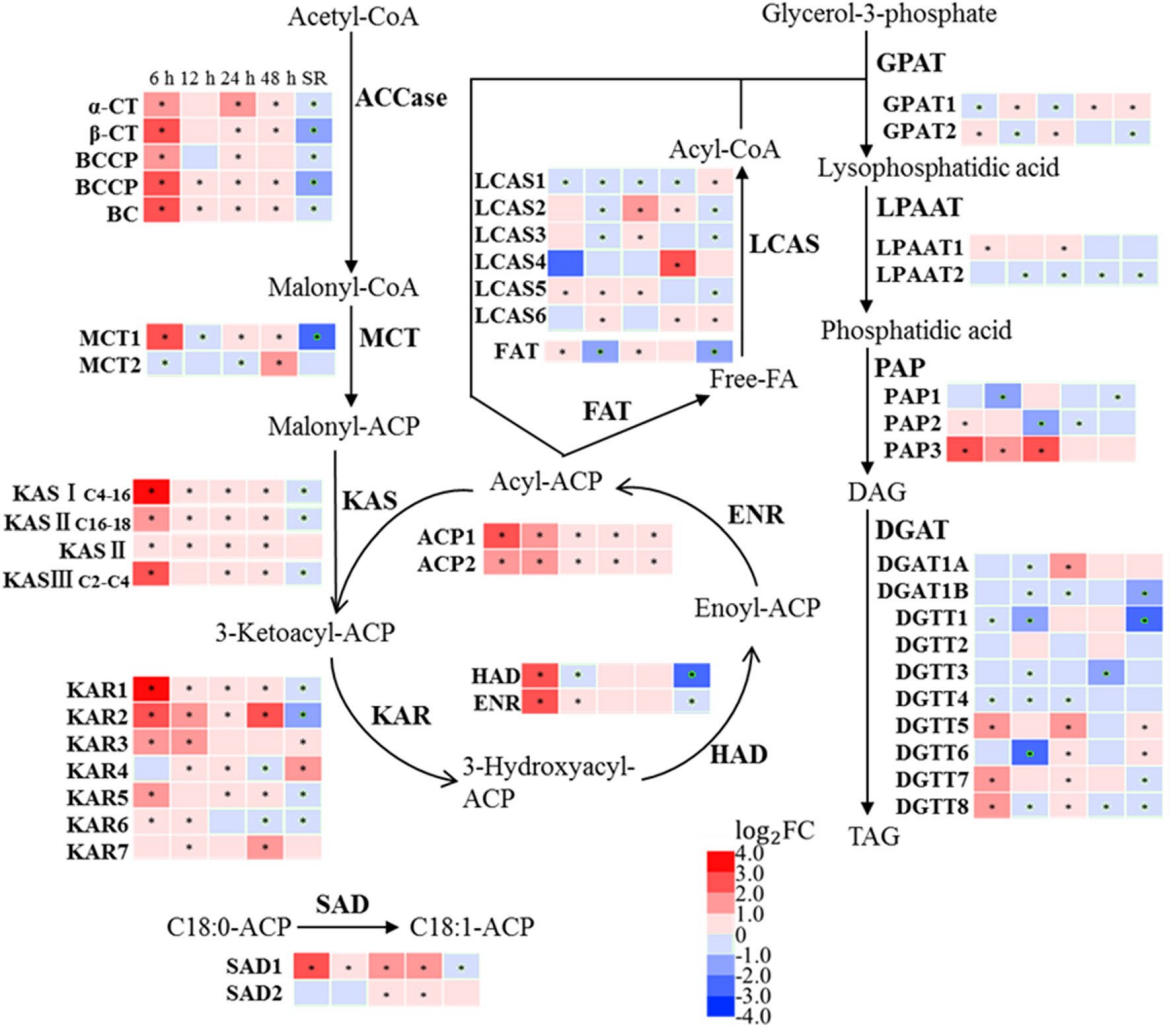
Transcriptional regulation of FA and TAG biosynthesis in response to SS and SR. The asterisk indicates Adjusted *p* value (*p*_adj_) ≤ 0.05. ACCase, acetyl-CoA carboxylase; CT, carboxyltransferase subunit; BCCP, biotin carboxyl carrier protein; BC, biotin carboxylase; MCT, malonyl-CoA:Acyl carrier protein transacylase; KAS, 3-oxoacyl-[acyl-carrier-protein] synthase; KAR, 3-oxoacyl-[acyl-carrier protein] reductase; HAD, 3-hydroxyacyl-[acyl-carrier-protein] dehydratase; ENR, enoyl-[acyl-carrier protein] reductase; SAD, Acyl-[acyl-carrier-protein] desaturase; FAT, acyl carrier protein thioesterase; LCAS, long-chain acyl-CoA synthetase; GPAT, glycerol-3-phosphate acyltransferase; LPAAT, 1-acyl-sn-glycerol-3-phosphate acyltransferase; PAP, phosphatidate phosphatase; DGAT, Diacylglycerol *O*-acyltransferase, type I; DGTT, Diacylglycerol *O*-acyltransferase, type II

Considering the potential of using *C. zofingiensis* for co-production of TAG and astaxanthin, the genes encoding key enzymes of the TAG and carotenoids’ biosynthesis pathway were identified (Additional file 4: Table S2), and their transcriptional level at each time point was analyzed (Fig. 6).

Fatty acids (FAs) are required as the substrates for TAG production, and hence the expression of the key genes in the FA biosynthesis pathways was analyzed first. Acetyl-CoA is the precursor in the de novo FA biosynthesis pathway, and acetyl-CoA carboxylase (ACCase) produces malonyl-CoA which is used in fatty acid synthesis and elongation of fatty acids [29]. Subunits of ACCase [the biotin carboxylase (BC), the biotin carboxyl binding protein (BCCP), and α- and ß-carboxyltransferases (α- and ß-CT)] were all significantly up-regulated within 6 h of SS. Most identified gene orthologs encoding enzymes in De novo FA synthesis pathway were up-regulated, including 3-ketoacyl-ACP synthase (KAS), 3-ketoacyl-ACP reductase (KAR), 3-hydroxyacyl-ACP-dehydratase (HAD), enoyl-ACP reductase (ENR) and stearoyl-ACP desaturase (SAD), among which the transcription level of KASI (~ 14.0-fold), KAR1 (~ 10.9-fold) and KAR2 (~ 7.4-fold) was up-regulated most. After SR, the transcription level of all genes above resumed to the pre-SS level.

The biosynthesis of TAG is achieved by esterification of FAs to glycerol-3-phosphate (G3P) via the Kennedy pathway. This route of TAG biosynthesis is referred as the acyl-CoA dependent route, using diacylglycerol (DAG) and acyl-CoA as substrates, and diacylglycerol acyltransferase (DGAT) as catalyst [30]. The acyl-CoA independent route is catalyzed by phospholipid:diacylglycerol acyltransferase (PDAT) using DAG and an acyl chain from glycerolipid molecules (phospholipid, galactolipid or DAG) as substrates [30, 31]. Among the genes encoding enzymes in the Kennedy pathway (Fig. 6), the transcription level of glycerol-3-phosphate acyltransferase (GPAT) and lysophosphatidate acyltransferase (LPAAT) remains unchanged throughout the test, while the expression of *PAP3* (*Cz16g11240*), one of the three genes encoding phosphatidate phosphatase (PAP), was strongly up-regulated (~ 7.4-fold). Although the other two PAP coding genes (*PAP1*, *Cz05g23060* and *PAP2*, *Cz10g16040*) were slightly down-regulated at 12 h or 24 h, the abundance of their transcripts was relatively low (< 10% of *PAP3*) at all sampled time points; hence, the impact of such transcriptional adjustment might be insignificant to the biosynthesis of TAG. Diglyceride acyltransferase (DGAT) is the enzyme catalyzing the final step of the TAG biosynthesis pathway; among the 10 DGAT isoforms, the expressions of *DGAT1A*, *DGTT5*, *DGTT7* and *DGTT8* were significantly up-regulated under SS, while that of *DGTT1*, *DGTT3* and *DGTT6* were down-regulated. Differential expression of the DGAT homologs has been reported previously, and is speculated to link with the functional divergence of these isomeric proteins [32]. The impact of SS on the acetyl-CoA independent pathway was also assessed; the expression level of the gene encoding PDAT was unaffected. (Additional file 4: Table S2).

### The transcriptional impact of SS on astaxanthin biosynthesis

**Fig. 7:**
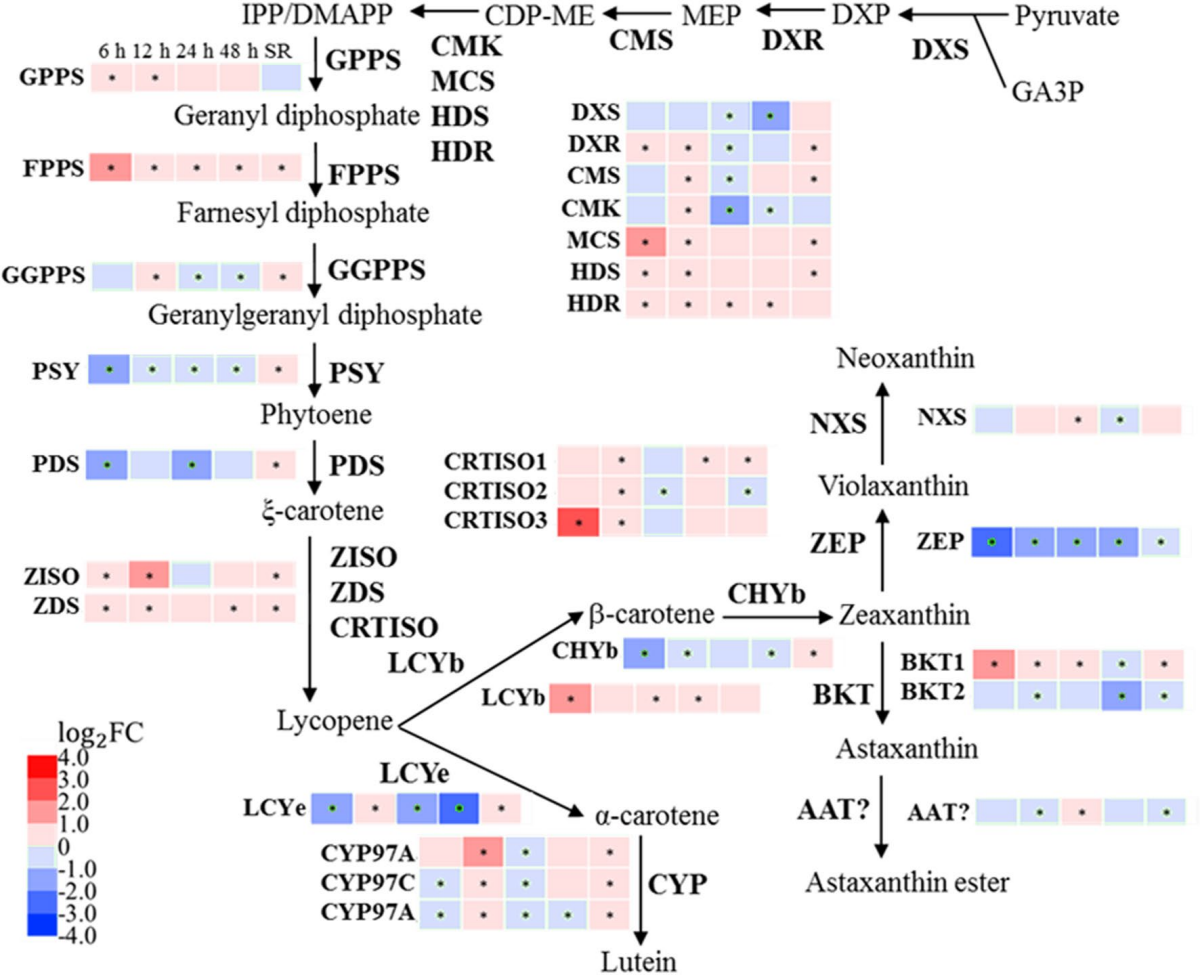
Transcriptional regulation of carotenoids biosynthesis in response to SS and SR. The asterisk indicates Adjusted *p* value (*p*_adj_) ≤ 0.05. DXS, 1-deoxy-d-xylulose 5-phosphate synthase; DXR, 1-deoxy-d-xylulose 5-phosphate reductoisomerase; CMS, 2-C-methyl-d-erythritol 4-phosphate cytidylyltransferase; CMK, 4-diphosphocytidyl-2-C-methyl-d-erythritol kinase; MCS, 2-C-methyl-d-erythritol 2,4-cyclodiphosphate synthase; HDS, 4-hydroxy-3-methylbut-2-en-1-yl diphosphate synthase; HDR, 4-hydroxy-3-methylbut-2-enyl diphosphate reductase; GPPS, geranyl diphosphate synthase; FPPS, farnesyl diphosphate synthase; GGPPS, geranylgeranyl diphosphate synthase; PSY, phytoene synthase; PDS, phytoene desaturase; ZISO, zeta-carotene isomerase; ZDS, zeta-carotene desaturase; CRTISO, carotenoid isomerase; LCYe, lycopene epsilon cyclase; LCYb, lycopene beta cyclase; CYP, cytochrome P450 beta hydroxylase; CHYb, beta-carotene hydroxylase; BKT, beta-carotene ketolase; AAT, long-chain-alcohol O-fatty-acyltransferase; ZEP, zeaxanthin epoxidase; NXS, neoxanthin synthase. GA3P, glyceraldehyde 3-phosphate; DXP, 1-deoxy-d-xylulose 5-phosphate; MEP, 2-C-methyl-d-erythritol 4-phosphate; CDP-ME, 4-(cytidine 5-diphosho)-2-C-methyl-d-erythritol; IPP, isopentenyl pyrophosphate; DMAPP, dimethylallyl pyrophosphate

Green microalgae use the MEP (2-C-methylerythritol 4-phosphate) pathway to produce isopentenyl pyrophosphate (IPP) and dimethylallyl pyrophosphate (DMAPP) as the precursors for the carotenoids biosynthesis, and the genes of all enzymes in the MEP pathway have been identified in *C. zofingiensis* [9, 33]. Among the genes in the MEP pathway, only that of 2-C-methyl-d-erythritol 2,4-cyclodiphosphate synthase (MCS) was up-regulated at early SS (Fig. 7). The initial step of carotenoid biosynthesis requires three IPP molecules sequentially condensed into DMAPP to form geranylgeranyl diphosphate (GGPP), and then two GGPPs are catalyzed into the first carotenoid phytoene, which is then converted into lycopene by sequential desaturation and isomerization. Among these steps, only the transcription of farnesyl diphosphate synthase (FPPS) and carotenoid isomerase (CRTISO3) was transiently up-regulated within 6 h under SS, while that of phytoene synthase (PSY) and phytoene desaturase (PDS) were down-regulated (Fig. 7). Lycopene can be either catalyzed into α-carotene by Lycopene epsilon cyclase (LCYe) or β-carotene by Lycopene beta cyclase (LCYb). Under SS, the transcription of LCYe was strongly down-regulated at 6 h, 24 h and 48 h; but that of LCYb was up-regulated at 6 h, which indicated that the flux of the terpenoid backbone was directed toward the β-carotene biosynthesis pathway. Beta-carotene hydroxylase (CHYb) and beta-carotene ketolase (BKT) are responsible for the final conversion of β-carotene into astaxanthin. The transcript abundance of BKT1, the dominant BKT homolog [33], was substantially increased to 2.45-fold under SS. The transcription level of zeaxanthin epoxidase (ZEP) was also down-regulated, indicating cells favor astaxanthin biosynthesis through suppression of competing pathways under SS. After SR, the transcription level of LCYb and BKT1 reversed to a low expressional level, and the down-regulated LCYe and ZEP were also reversed.

### The transcriptional impact of SS on central carbon metabolism

**Fig. 8:**
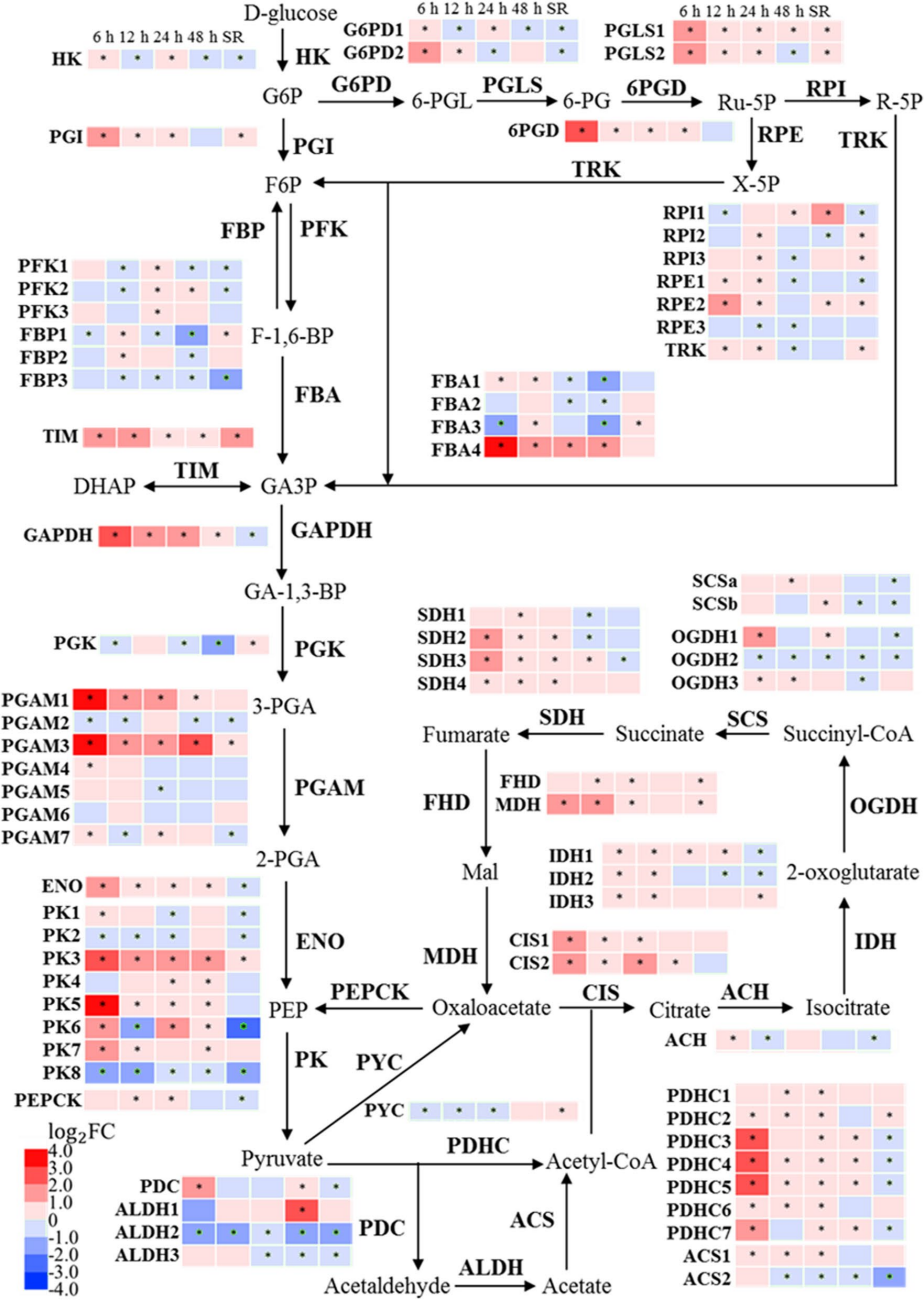
Transcriptional regulation of central carbon metabolism in response to SS and SR. The asterisk indicates Adjusted *p* value (*p*_adj_) ≤ 0.05. HK, hexokinase; PGI, glucose-6-phosphate isomerase; FBP, fructose-1,6-bisphosphatase; FBA, fructose-bisphosphate aldolase; TIM, triosephosphate isomerase; GAPDH, glyceraldehyde 3-phosphate dehydrogenase; PGK, phosphoglycerate kinase; PGAM, phosphoglycerate mutase, 2,3-bisphosphoglycerate; ENO, enolase; PK, pyruvate kinase; G6PD, glucose-6-phosphate 1-dehydrogenase; PGLS, 6-phosphogluconolactonase; 6PGD, 6-phosphogluconate dehydrogenase; RPI, ribose 5-phosphate isomerase; RPE, ribulose-phosphate 3-epimerase; TRK, transketolase; PFK,; PYC, pyruvate carboxylase; CIS, citrate synthase; ACH, aconitate hydratase; IDH, isocitrate dehydrogenase; OGDH, 2-oxoglutarate dehydrogenase; SCS, succinyl-CoA synthetase; SDH, succinate dehydrogenase; FHD, fumarate hydratase; MDH, malate dehydrogenase; PEPCK, phosphoenolpyruvate carboxykinase; PDHC, pyruvate dehydrogenase complex; ACS, acetyl-CoA synthetase; ALDH, aldehyde dehydrogenase; PDC, pyruvate decarboxylase. G6P, glucose-6-phosphate; F6P, fructose-6-phosphate; F-1,6-BP, fructosel 1,6-bisphosphate; GA3P, glyceraldehyde 3-phosphate; GA-1,3-BP, glyceraldehyde 1,3-biphosphate; PGA, phosphoglycerate; PEP, phosphoenolpyruvate; 6-PGL, 6-phosphogluconolactone; 6-PG, 6-phosphogluconate; Ru-5P, ribulose-5-phosphate; R-5P, ribose 5-phosphate; X-5P, xylulose 5-phosphate

As carbon partitioning is an important regulatory mechanism that influences the dynamics of lipid and carotenoid biosynthesis, the expression patterns of the genes involved in central carbon metabolism were investigated. Under SS, the expressions of most genes in the oxidative pentose phosphate (OPP) pathway were elevated, including that of glucose-6-phosphate dehydrogenase (G6PD), 6-phosphogluconolactonase (PGLS), 6-phosphogluconate dehydrogenase (6PGD), ribose 5-phosphate isomerase (RPI) and ribulose-phosphate 3-epimerase (RPE) (Fig. 8). Notably, G6PD and 6PGD catalyze the NADPH-producing steps, which could be influential to the FA and astaxanthin biosynthesis pathway. The expression of genes belonging to the tricarboxylic acid (TCA) cycle was also up-regulated under SS, including citrate synthase (CIS), 2-oxoglutarate dehydrogenase (OGDH), succinate dehydrogenase (SDH) and malate dehydrogenase (MDH). Thus, it is likely that more energy was produced from the TCA cycle to support the biosynthesis of TAG and astaxanthin. The transcript abundance of up-regulated genes in the OPPP pathway and the TCA cycle was resumed to pre-SS level after SR.

The expression pattern of genes involved in glycolysis and gluconeogenesis was more complex under SS. The pyruvate generated from the glycolysis pathway is the essential precursors for lipid and carotenoids’ biosynthesis [34]; therefore, the transcriptional regulation of the glycolysis pathway was analyzed (Fig. 8). The transcript abundance of enolase (ENO), which catalyzes the conversion of 2-phosphoglycerate (2-PGA) to phosphoenolpyruvate (PEP), was increased during SS. The expression levels of four of the eight putative pyruvate kinase (PK) responsible for the downstream catalysis to produce pyruvate, namely PK3, PK5, PK6 and PK7, were also up-regulated. After SR, the expression level of ENO, PK3, PK5 and PK7 was comparable with the control group, and PK6 was down-regulated. In summary, the up-regulation of the key genes in the glycolysis pathway could result in increased availability of pyruvate for lipid and carotenoid biosynthesis under SS.

## Discussion

### The physiological acclimation of *C. zofingiensis* to sulfur-starvation

To survive the dynamic environment, microalgae have developed sophisticated molecular mechanisms to respond environmental stimuli and acclimate to prolonged stress. While the cellular responses to N starvation have been well characterized through multi-omics methods, that of S starvation is yet unclear. In this study, phenotypical and morphological changes on *C. zofingiensis* were monitored, when cells were subjected to SS and the recovery process under SR. The photosynthetic system displayed severe damage during SS, as the chlorophyll content sharply decreased (Fig. 1c) and the thylakoid membranes were impaired (Fig. 2a). The TEM work in this study is consistent with the data from previous report that the abundance of the main lipid components of the thylakoid membranes, namely monogalactosyl diacylglycerol (MGDG), digalactosyl diacylglycerol (DGDG), sulfoquinovosyl diacylglycerol (SQDG) and phosphatidylglycerol (PG), was significantly decreased under SS [7]. In tandem to the observation that the transcription of many photosynthetic genes was also repressed at SS 6 h (Additional file 4: Fig. S6a), the photosynthetic activity in *C. zofingiensis* was inhibited under SS, which would have inevitably led to the growth arrest phenomenon as observed (Fig. 1d). After SR, the transcription of the photosynthesis-associated genes was rapidly up-regulated (Additional file 4: Fig. S6b), and the increase in chlorophyll content and the presence of the thylakoid membranes indicated the recovery of photosynthesis.

Under sulfur stress, the *C. zofingiensis* cells turned larger in size with altered cellular structure and biochemical composition (Fig. 2). Further analysis indicated that the accumulation of lipids such as FAs and astaxanthin was the result of redirecting carbon flux from protein biosynthesis toward fatty acids and carotenoids’ biosynthesis, whereas the starch metabolism seems not affected. The replenishment of sulfate effectively restored the phenotype and the gene expression pattern within 12 h, which demonstrated the reversible nature of the SS-induced metabolic responses.

Sulfur plays an important role in metabolism, for example the sulfur-containing amino acids methionine is required for protein biosynthesis and cysteine is an indispensable precursor of glutathione, which is crucial to cellular redox tuning [35]. Sulfur shortage caused apparent raise in ROS level (Fig. 1f). Importantly, ROS has been reported as signaling molecules for metabolic regulations [36]. The synthesis pathways of FAs and astaxanthin serve as electron sinks under stress conditions, and unsaturated fatty acids and astaxanthin are also antioxidants to mediate oxidative stress [37, 38]. In addition, it has been reported that supplementation of ROS could promote astaxanthin accumulation in *C. zofingiensis* [39]. Therefore, the enhanced ROS production in vivo could be an important factor to TAG and astaxanthin accumulation under the SS condition.

### The transcriptome dynamics of TAG and astaxanthin biosynthesis under SS

Acetyl-CoA is the precursor for FA synthesis, and thus the availability of cellular acetyl CoA plays an important role in TAG accumulation. In chloroplasts, there are two main routes for acetyl-CoA biosynthesis: one route is through decarboxylation of pyruvate catalyzed by pyruvate dehydrogenase complex (PDHC); and the other is via conversion of acetate to acetyl-CoA catalyzed by acetyl-CoA synthetase (ACS) [40]. In addition, a less-defined route, known as “pyruvate dehydrogenase (PDH) bypass”, which converts acetyl-CoA from pyruvate via sequentially reactions is catalyzed by aldehyde dehydrogenase (ALDH) and ACS [40]. In *C. zofingiensis*, four of seven PDHC genes, namely *PDHC3 (Cz03g08090)*, *PDHC4 (Cz01g37230)*, *PDHC5 (Cz05g28130)* and *PDHC7 (Cz07g16120)*, were strongly up-regulated at early SS (6 h) (Fig. 8), indicating PDHC could be one of the main enzymes contributes to the acetyl-CoA production during SS-induced TAG accumulation and potential target for genetic engineering. *ACS* was not up-regulated under SS, while under N starvation *ACS* also contributes to the production of acetyl-CoA [10]. Pyruvate is an important precursor for central carbon metabolism and the biosynthesis of lipid and astaxanthin. The elevated glycolysis pathway under SS increased availability of pyruvate for lipid and carotenoid production. Previous transcriptome analysis on glucose feeding experiments also showed increases in gene expression of nearly all genes involved in glycolysis, and hence, the increase in lipid biosynthesis under glucose supplementation condition might also be the results of increased pyruvate availability [17].

**Fig. 9:**
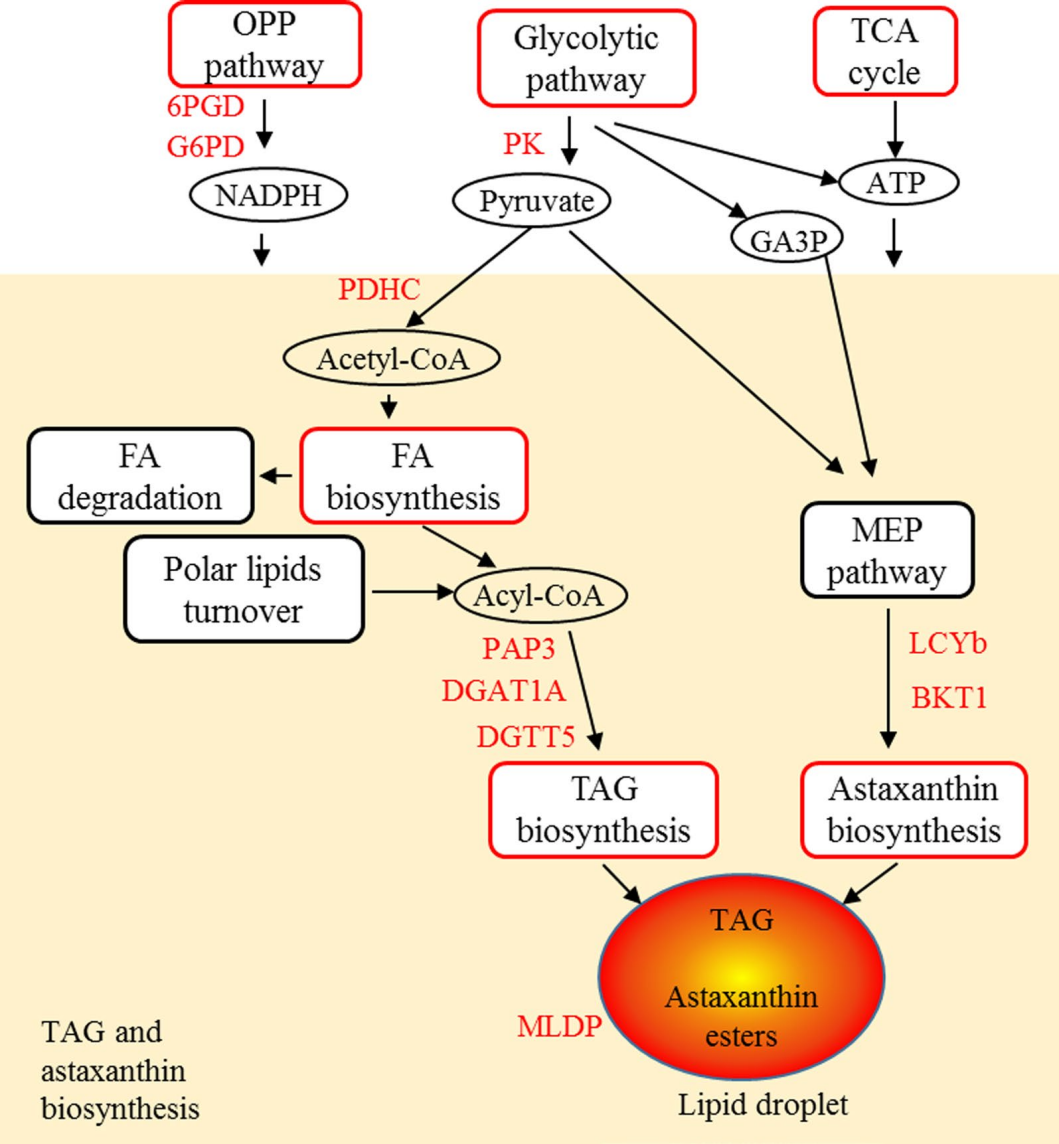
A hypothetical model for underlying mechanism of TAG and astaxanthin hyperaccumulation under SS stress. The red color of boxes and genes indicates up-regulation. The orange box represents TAG and astaxanthin biosynthesis pathway. OPP pathway, oxidative pentose phosphate pathway; 6PGD, 6-phosphogluconate dehydrogenase; G6PD, glucose-6-phosphate 1-dehydrogenase; TCA cycle, tricarboxylic acid cycle; GA3P, glyceraldehyde 3-phosphate; FA, fatty acid; PK, pyruvate kinase; PDHC, pyruvate dehydrogenase complex; PAP, phosphatidate phosphatase; DGAT, Diacylglycerol *O*-acyltransferase, type I; DGTT, Diacylglycerol *O*-acyltransferase, type II; LCYb, lycopene beta cyclase; BKT, beta-carotene ketolase; MLDP, major lipid droplet protein

Based on time-resolved transcriptome analysis, a hypothetical model of underlying mechanism of TAG and astaxanthin accumulation under SS was proposed (Fig. 9). The entire de novo FA synthesis pathway was up-regulated under SS, and was down-regulated under SR. Thus, it is likely that the de novo FA synthesis was indicated to be the major source of acyl substrates for TAG accumulation under SS. In TAG biosynthesis pathway, PAP3 and DGATs (DGAT1A, DGTT5, DGTT7 and DGTT8) could be the key rate-limiting enzymes for de novo TAG biosynthesis as the genes of which were up-regulated under SS. Moreover, it has been demonstrated that DGAT1A and DGTT5 had the highest activity among all ten CzDGATs [32], and thus the up-regulation of DGAT1A and DGTT5 could play an important role in the TAG accumulation. *PAP3*, *DGAT1A* and *DGTT5* were promising gene targets for genetic engineering. PAP3 and DGATs were predicted to be ER-targeted [10, 32], suggesting that TAG was mainly assembled in ER under SS. Under N starvation, more genes in TAG biosynthesis pathway were up-regulated including extrachloroplastic GPAT2, LPAAT2 and chloroplastic LPAAT1 [10], which may account for the higher TAG content under N starvation (about 28.6% higher) than SS [7]. In addition, the expression of the major lipid droplet protein (MLDP) and plastid galactoglycerolipid degradation 1 (PGD1) were up-regulated during SS (Additional file 4: Table S2). As MLDP is associated with maintaining the size of lipid droplets (LDs) [41, 42], the up-regulation of *CzMLDP* (~ 7.41-fold) (Additional file 4: Table S2) could be a major factor for the increase in cellular lipid droplets under SS. CrPGD1 in *C. reinhardtii* was previously reported to be up-regulated under nitrogen starvation and involved in MGDG turnover for TAG synthesis [43]. In this study, the expression of CzPGD1, the homologous enzyme of CrPGD1, was fourfold increased at 6 h, 24 h and 48 h, and the transcription level of which was substantially reduced under SR (Additional file 4: Table S2); hence, the activity of CzPGD1 could correlate to the TAG accumulation.

Despite the increase in the cellular pyruvate pool under SS, the expression of most enzymes of the MEP pathway did not show significant change. The biosynthesis of astaxanthin requires the terpenoid backbones at two branching points. (1) The down-regulation of *LCYe* and up-regulation of *LCYb* redirected the lycopene to β-carotene, and (2) induction of *BKT1* and repression of *ZEP* reallocated the zeaxanthin into astaxanthin (Fig. 7), which represented the suppression of competing pathway and enhancement of the carbon flux toward astaxanthin biosynthesis. Consistently, the astaxanthin content was increased at the expense of β-carotene, indicating the redirection of precursors toward the astaxanthin. A similar expression pattern of *LCYe* and *ZEP* has also been observed in the glucose-induced astaxanthin accumulation experiment [17, 33]. In addition, the up-regulation of *LCYb* and *BKT1* was also observed under N starvation and glucose feeding [16, 17]. Therefore, *LCYb* and *BKT1* could be gene targets for pulling precursor toward astaxanthin by genetic engineering. And furthermore, suppression of competing pathways via genetic engineering would improve precursor supply and increase target compounds [44]. Compared with SS, the expression of *CHYb* was up-regulated under N starvation but not changed under SS, and *BKT1* was more up-regulated by N starvation [16], which may be the reason that more astaxanthin was accumulated under SS.

Apart from the substrate molecules, the FA and astaxanthin synthesis requires NADPH and ATP to provide reducing power and energy. For example, the biosynthesis of C18:1 FA needs 19 NADPH molecules as 18 are required to polymerize the C18 acyl chain, and one for the subsequent desaturation [45]. The MEP pathway also requires NADPH to synthesis the terpenoid backbone for carotenoids [34]. In phototrophic microalgae, the photosynthesis pathway generates NADPH from the linear electron chain [46]. However, because the thylakoid membrane was severely degraded and the chlorophyll content was dramatically reduced, the photosynthesis pathway was unlikely the main source of NADPH and ATP for the biosynthesis of FAs and astaxanthin under SS. Another source of cellular NADPH is the OPP pathway, which generates two NADPH molecules for each molecule of glucose-6-phosphate catalyzed. Overexpression of G6PD could elevate the NADPH content and consequently enhanced the lipid biosynthesis in microalgae [47, 48]. The OPP pathway was elevated under SS, and it has also been associated with nitrogen starvation-induced FAs and astaxanthin accumulation in *C. zofingiensis* [10]. Thus, the OPP pathway is likely to be the main source of NADPH under stress conditions, and the increasing reductant pool promotes the lipid and astaxanthin production.

## Conclusion

SS leads to a series of phenotypical responses in *C. zofingiensis*, including growth inhibition, TAG and astaxanthin accumulation, cellular structural alteration and metabolic flux reallocation. The time-resolved transcriptome analysis of *C. zofingiensis* indicated that the accumulation of TAG and astaxanthin under SS could be associated with elevated glycolysis and the OPP pathway, which produce the pyruvate and NADPH required for FAs and carotenoids biosynthesis. Key genes in de novo fatty acids biosynthesis pathway, including *ACCase*, *KAS*, *KAR*, *HAD* and *ENR*, were significantly up-regulated to provided acyl chain for TAG, and *PAP3* and *DGATs* (especially *DGTT5*) were key genes up-regulated in TAG assembly pathway. Moreover, LCYb and BKT1 played a role in diverting terpenoid backbones into astaxanthin accumulation along with the suppression of competing pathways catalyzed by LCYe and ZEP. Physiological and transcriptomic changes were reversed after SR. This study provided valuable information for understanding TAG and astaxanthin accumulation induced by sulfur deprivation, and identified a number of genes involved in TAG and astaxanthin biosynthesis as promising targets for metabolic engineering.

## Materials and methods

### Strain and culture conditions

*Chromochloris zofingiensis* (strain ATCC 30412, from the American Type Culture Collection) was cultured in Kulh medium using 250-mL column (3-cm diameter) photoreactor according to our previous study [7]. Cultivation conditions were 25 °C, constant illumination of 80 μE m^−2^ s^−1^ and aeration of 1.5% CO_2_ enriched air in all experiments. For sulfur-starvation (SS), cells in logarithmic phase were resuspended in modified Kulh medium in which MgSO_4_ was replaced by MgCl_2_ in equal molar concentration. At the second stage for sulfur-replenishment (SR), 1 mM Na_2_SO_4_ was added into culture.

### Measurement of biomass, cell number and chlorophyll content

The biomass dry weight was determined by filtration through a pre-dried and pre-weighted Whatman GF/C filter paper (1.2 μm pore size), which was dried at 80 °C in vacuum oven. Cell number was counted using a hemocytometer. Chlorophyll in fresh algal cells was extracted with 5 mL methanol by grinding, and centrifuged (13,000 rpm, 5 min) before measurement. By measuring the optical density respectively at 665 nm, 652 nm and 750 nm with a spectrophotometer, chlorophyll concentration was calculated with the following equation [49]. Absorbencies at 652 nm, 665 nm and 480 nm were corrected by subtracting absorbency at 750 nm.

$$ \left[ {{\text{Chlorophyll}}\;{\text{a}}} \right]{\text{mg}}\;{\text{L}}^{ - 1} = 16.519 \times A_{665} - 8.0962 \times A_{652} $$

$$ \left[ {{\text{Chlorophyll}}\;{\text{b}}} \right]{\text{mg}}\;{\text{L}}^{ - 1} = 27.4405 \times A_{652} - 12.1688 \times A_{665} $$

### ROS level analysis

ROS levels were measured using the ROS assay kit (Beyotime Institute of Biotechnology, China). 10^7 cells were collected and incubated with 10 μM 2′,7′-dichlorodihydrofluorescein diacetate (DCF-DA) for 20 min, and washed three times to remove redundant probes. Nonfluorescent DCF-DA could diffuse into cells and oxidized by ROS into highly fluorescent 2′,7′-dichlorofluorescein (DCF). The fluorescent intensity of DCF was determined at 485 nm excitation and 520 nm emission by a fluorescence microplate reader (Tecan Infinite F200 Pro, USA). Finally, ROS fold change was expressed as the ratio of ROS level of SS or SR samples to that of control samples.

### Transmission electron microscopy (TEM) and measurement of protein and starch content

Cells were collected and fixed overnight in 2.5% glutaraldehyde buffer (electron microscope purity) at 4 °C. After washing, fixed mixture of osmium tetroxide and potassium ferricyanide was added to the sample for post-fixation at room temperature for 2 h. Gradient dehydration on the sample was performed using 30%–50%–70%–90%–95%–100% alcohol for 10 min per step. After replacing the alcohol with acetone, resin infiltration was performed on the rotary mixer. Then, epoxy resin with 1.5–2% of the catalyst was injected into the embedding mold, and the sample was placed in the resin of the embedding mold. Polymerization in an oven was conducted at 35° C for 12 h, then 45° C for 12 h, and then 60° C for 24 h. Sample frozen and specimen preparation were carried out according to previous study [17]. After stained with acetic acid glaze and lead citrate, subcellular structure was observed using TEM (JEOL JEM1200EX) operated at 120 kV.

Lyophilized microalgal cells were grinded and lysed by extraction solution [containing 50 mM Tris (pH 8.1), 1% Sodium dodecyl sulfate (SDS), sodium pyrophosphate, β-glycerophosphate, sodium orthovanadate, sodium fluoride, EDTA, and phenylmethanesulfonyl fluoride (PMSF)]. The supernatant was collected for protein content determination using BCA assay kit (Beyotime Institute of Biotechnology, China) by measuring the optical density at 562 nm with a spectrophotometer.

For starch content analysis, lyophilized cells were grinded and removed soluble sugar by incubation at 80 °C for 30 min with 80% ethanol solution. The mixture was cooled down and centrifuged to collect the pellets. The pellet was then boiled to digest the resistant starch, and amylase (Solarbio Science and Technology, China) was added to the mixture for the hydrolysis of starch. After hydrolysis, anthrone and sulfuric acid were used to incubate with released glucose at 95 °C for 10 min, and the optical density at 562 nm was determine to calculate the starch content of each sample as equivalents to the glucose.

### Lipid extraction and analysis

Lyophilized algal cells were grinded with liquid nitrogen, and total lipids were extracted by a solvent mixture of chloroform/methanol/0.75% aqueous NaCl solution (2:1:0.75, by volume). The chloroform layer containing lipid was extracted for lipid analysis. For total lipid quantification, lipid in chloroform was directly transesterificated with 1% (v/v) sulfuric acid in methanol at 85 °C for 2 h, and analyzed using gas chromatography–mass spectrometry (GC–MS) equipped with a DB-WAX capillary column (30 m × 0.25 mm × 0.25 μm) (Agilent, CA, USA). Heptadecanoic acid (C17:0, Sigma-Aldrich) was used as the internal standard. Helium was used as the carrier gas with the flow rate of 1.2 mL/min. The ion temperature and interface temperature were 200 °C and 240 °C, respectively. Samples were injected in split mode (5:1 split ratio) at an oven temperature of 45 °C with an injection volume of 1 μL. The oven temperature was raised to 150 °C at a rate of 15 °C min^−1^, then to 240 °C at a rate of 6 °C min^−1^ and held for 6 min. For TAG quantification, TAG was separated on a Silica gel 60 TLC plate (EMD Chemicals, Merck, Germany) using a mixture of hexane/tert-butylmethyl ether/acetic acid (80/20/2, by volume) as the mobile phase, then transesterificated and analyzed using above method.

### Carotenoids’ extraction and analysis

Pigment was extracted from lyophilized algal cells by acetone after cells were grinded with liquid nitrogen. Astaxanthin and β-carotene were analyzed on HPLC (Waters, Milford, MA, USA) equipped with a Shiseido CAPCELL PAK C18 5 μm column (4.6 × 250 mm) and a 2998 photodiode array detector (Waters, Milford, MA, USA). The gradient was as below: the gradient was started from 100% solvent B (acetonitrile/methanol/water, 84:2:14, by volume); from 0 to 15 min, solvent A (ethyl acetate) and solvent C (methanol) linearly increased from 0 to 20% and from 0 to 80%, respectively, followed by a linear gradient to 32% solvent A and 68% solvent C from 15 to 20 min; and hold on this gradient for 10 min, then linearly to 80% solvent A and 20% solvent C from 30 to 50 min; finally turned back to 100% solvent B and hold on for 5 min. The flow rate was 0.8 mL min^−1^.

### RNA extraction and RNA-seq analysis

Total RNA was extracted using the plant RNA extraction kit (TaKaRa, Tokyo, Japan), and contaminating DNA was removed with RNase-free DNase I (TaKaRa, Tokyo, Japan). RNA concentration and quality were analyzed by Agilent 2100 Bio analyzer. After purification of poly-A containing mRNA molecules using poly-T oligo-attached magnetic beads, the mRNA was fragmented into small pieces and copied into first strand cDNA, followed by second strand cDNA synthesis using DNA polymerase I and RNase H. These cDNA fragments then had the addition of a single ‘A’ base and subsequent ligation of the adapter. The products were purified and enriched with PCR amplification. The PCR yield was quantified by Qubit, and samples were pooled together to make a single-strand DNA circle (ssDNA circle) to form the final library. DNA nanoballs (DNBs) were generated with the ssDNA circle to enlarge the fluorescent signals at the sequencing process. The DNBs were loaded into the patterned nanoarrays and single-end read of 50 bp was read through on the BGISEQ-500 platform (BGI, China) for the following data analysis study. For this step, the BGISEQ-500 platform combined the DNA nanoball-based nanoarrays and stepwise sequencing using Combinational Probe-Anchor Synthesis Sequencing Method.

### Differential gene expression analysis

Clean reads were obtained by filtering the low-quality reads (more than 20% of the bases qualities are lower than 10), reads with adaptors and reads with unknown bases (N bases more than 5%) to get the clean reads, then mapped into reference genome [9] using HISAT (Hierarchical Indexing for Spliced Alignment of Transcripts) software and into reference genes using Bowtie2 software. RSEM software was used to calculate gene expression level from RNA-seq data. R software was used to perform statistics: Pearson correlation between all samples was calculated using cor; and PCA analysis was performed with all samples using princomp. Finally, we identify DEGs (differential expressed genes) between samples and do clustering with DEseq2 software using the parameters as Fold Change ≥ 2.00 and Adjusted *p* value (*P*_adj_) ≤ 0.05. The RNA-seq data were deposited in the Gene Expression Omnibus under accession number GSE130454.

### Statistical analysis

All the experiments were conducted in at least three biological replicates to ensure the reproducibility. Experimental results were expressed as mean value ± SD. The statistical significance of the results was tested by *t* test.

## Supplementary information

**Additional file 1.** Gene expression level in transcriptome of *C. zofingiensis*.

**Additional file 2.** KEGG pathway enrichment and GO term enrichment.

**Additional file 3.** The top 100 genes having highest differential expression in SS 6 h group.

**Additional file 4.: Fig. S1.** Carotenoids contents after 4-days culture. (a) Astaxanthin forms composition (b) α-carotene content (c) total carotenoids content. **Fig. S2.** Volcano plot of DEGs. (a) SS-6 h (b) SS-12 h (c) SS-24 h (d) SS-48 h (e) SR-12 h. **Fig. S3.** KEGG pathway classification and of DEGs. (a-b) KEGG pathway classification in SS-6 h (0 h as control) and SR-12 h (SS-48 h as control) (c-d) KEGG pathway functional enrichment in SS-6 h (0 h as control) and SR-12 h (SS-48 h as control). **Fig. S4.** Transcriptional regulation at SS-6 h (0 h as control). (a) KEGG pathway of “photosynthesis-antenna protein” (b) KEGG pathway of “proteasome”. The red and blue boxes indicate up-regulation and down-regulation respectively. **Fig. S5.** Hierarchical cluster analysis of DEGs responding to both SS and SR in enriched KEGG pathways. **Fig. S6.** Transcriptional regulation in KEGG pathway of “photosynthesis”. (a) SS-6 h (0 h as control) (b) SR-12 h (SS-48 h as control). The red and blue boxes indicate up-regulation and down-regulation respectively. **Fig. S7.** RT-PCR results of *β*-*CT* (a), *DGTT5* (b) and *BKT1* (c) at early SS. **Table S1.** Reads number and mapping ratio of RNA profiling. **Table S2.** Expression patterns of genes involved in TAG, astaxanthin biosynthesis and central carbon mechanism. **Table S3.** Fatty acid composition of TAG and TFA after 4-days culture. **Table S4.** ROS abundance in *C. zofingiensis* under different status.

## Data Availability

All data generated or analyzed during this study are included in this published article (and its additional files).

## Abbreviations

ACCase: Acetyl-CoA carboxylase
BKT1: Beta-carotene ketolase
DEGs: Differentially expressed genes
DGAT: Diacylglycerol acyltransferase
FA: Fatty acid
LCYb: Lycopene beta cyclase
OPP: Oxidative pentose phosphate
ROS: Reactive oxygen species
SR: Sulfur-replenishment
SS: Sulfur-starvation
TAG: Triacylglycerol
TCA: Tricarboxylic acid
TEM: Transmission electron microscopy
TFA: Total fatty acids
