# Supplementary material for: Time‑resolved transcriptome analysis during transitions of sulfur nutritional status provides insight into triacylglycerol (TAG) and astaxanthin accumulation in the green alga Chromochloris zofingiensis
Source: Biotechnol Biofuels. 2020 Jul 17;13:128. doi: 10.1186/s13068-020-01768-y (PMC7367374; doi:10.1186/s13068-020-01768-y)
Supplement: Supplementary file 4 — Additional file 4.: Fig. S1. Carotenoids contents after 4-days culture. (a) Astaxanthin forms composition (b) α-carotene content (c) total carotenoids content. Fig. S2. Volcano plot of DEGs. (a) SS-6 h (b) SS-12 h (c) SS-24 h (d) SS-48 h (e) SR-12 h. Fig. S3. KEGG pathway classification and of DEGs. (a-b) KEGG pathway classification in SS-6 h (0 h as control) and SR-12 h (SS-48 h as control) (c-d) KEGG pathway functional enrichment in SS-6 h (0 h as control) and SR-12 h (SS-48 h as control). Fig. S4. Transcriptional regulation at SS-6 h (0 h as control). (a) KEGG pathway of “photosynthesis-antenna protein” (b) KEGG pathway of “proteasome”. The red and blue boxes indicate up-regulation and down-regulation respectively. Fig. S5. Hierarchical cluster analysis of DEGs responding to both SS and SR in enriched KEGG pathways. Fig. S6. Transcriptional regulation in KEGG pathway of “photosynthesis”. (a) SS-6 h (0 h as control) (b) SR-12 h (SS-48 h as control). The red and blue boxes indicate up-regulation and down-regulation respectively. Fig. S7. RT-PCR results of β-CT (a), DGTT5 (b) and BKT1 (c) at early SS. Table S1. Reads number and mapping ratio of RNA profiling. Table S2. Expression patterns of genes involved in TAG, astaxanthin biosynthesis and central carbon mechanism. Table S3. Fatty acid composition of TAG and TFA after 4-days culture. Table S4. ROS abundance in C. zofingiensis under different status. [file 13068_2020_1768_MOESM4_ESM.docx]

**Figures**

**
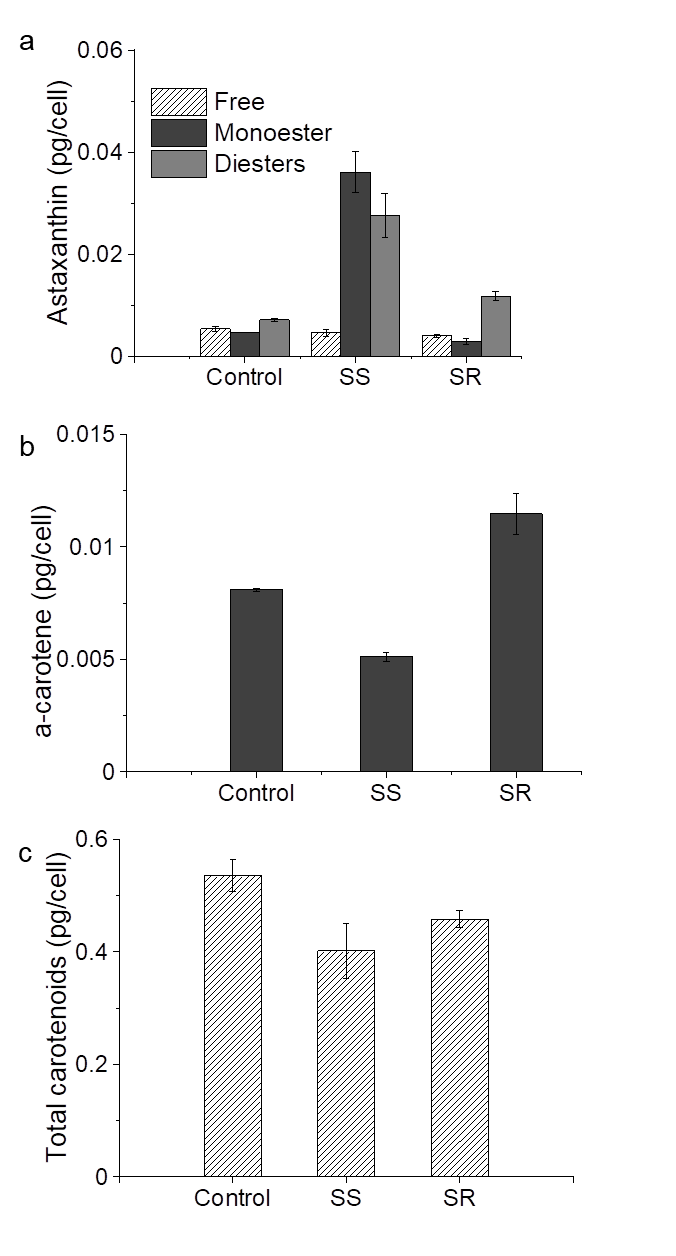
**

**Fig. S1. Carotenoids contents after 4-days culture. (a) Astaxanthin forms composition (b) α-carotene content (c) total carotenoids content.**


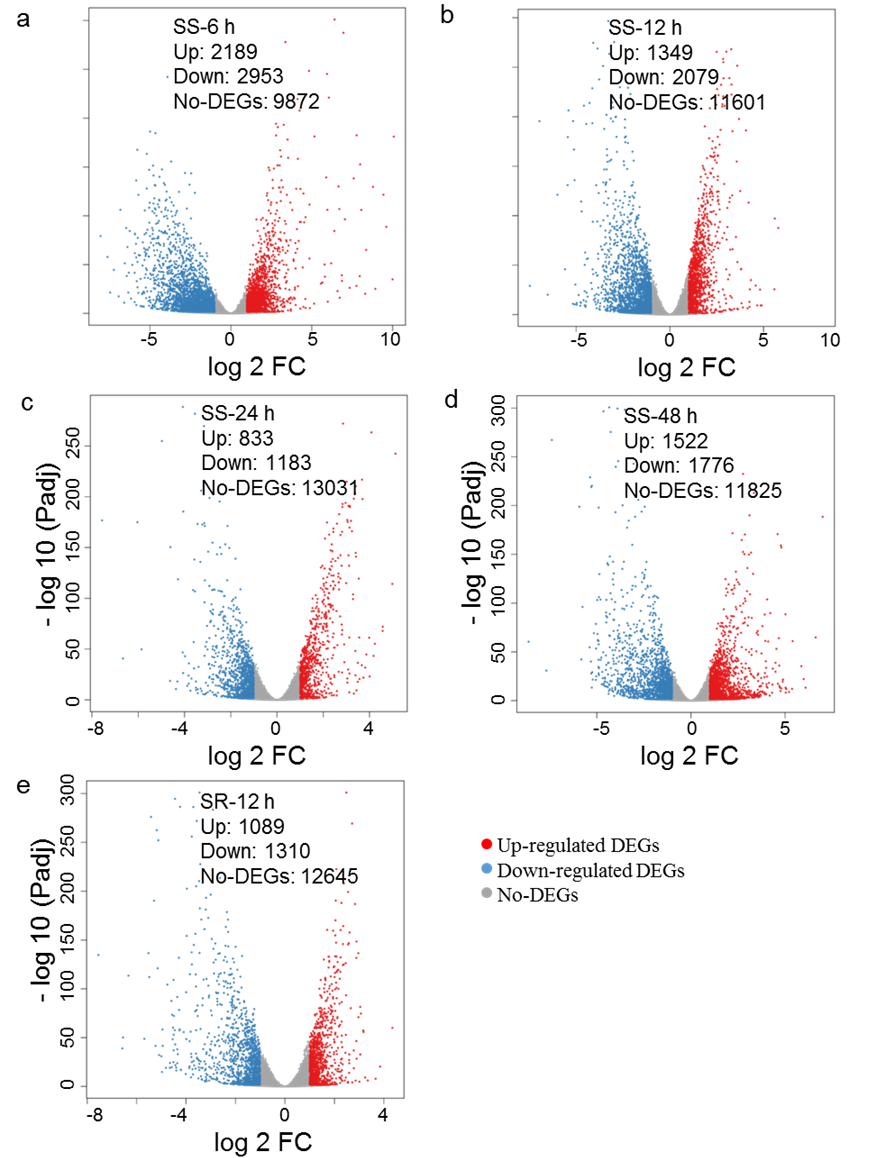


**Fig. S2. Volcano plot of DEGs. (a) SS-6 h (b) SS-12 h (c) SS-24 h (d) SS-48 h (e) SR-12 h.**


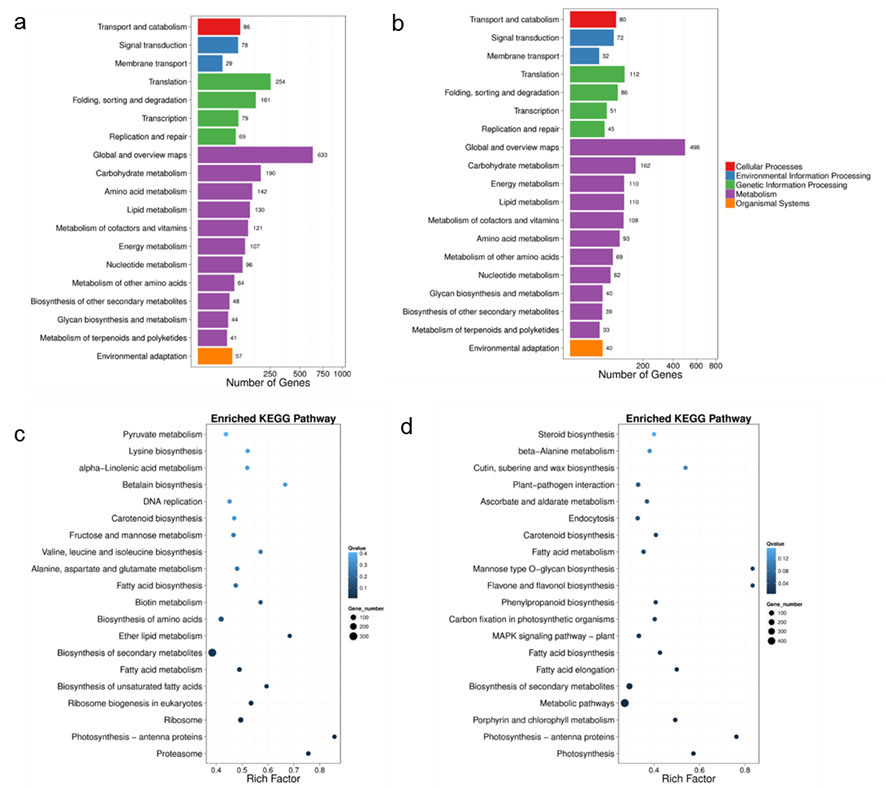


**Fig. S3. KEGG pathway classification and enrichment of DEGs. (a-b) KEGG pathway classification in SS-6 h (0 h as control) and SR-12 h (SS-48 h as control) (c-d) KEGG pathway functional enrichment in SS-6 h (0 h as control) and SR-12 h (SS-48 h as control). Rich factor indicates the ratio of DEGs to all genes in that pathway.**


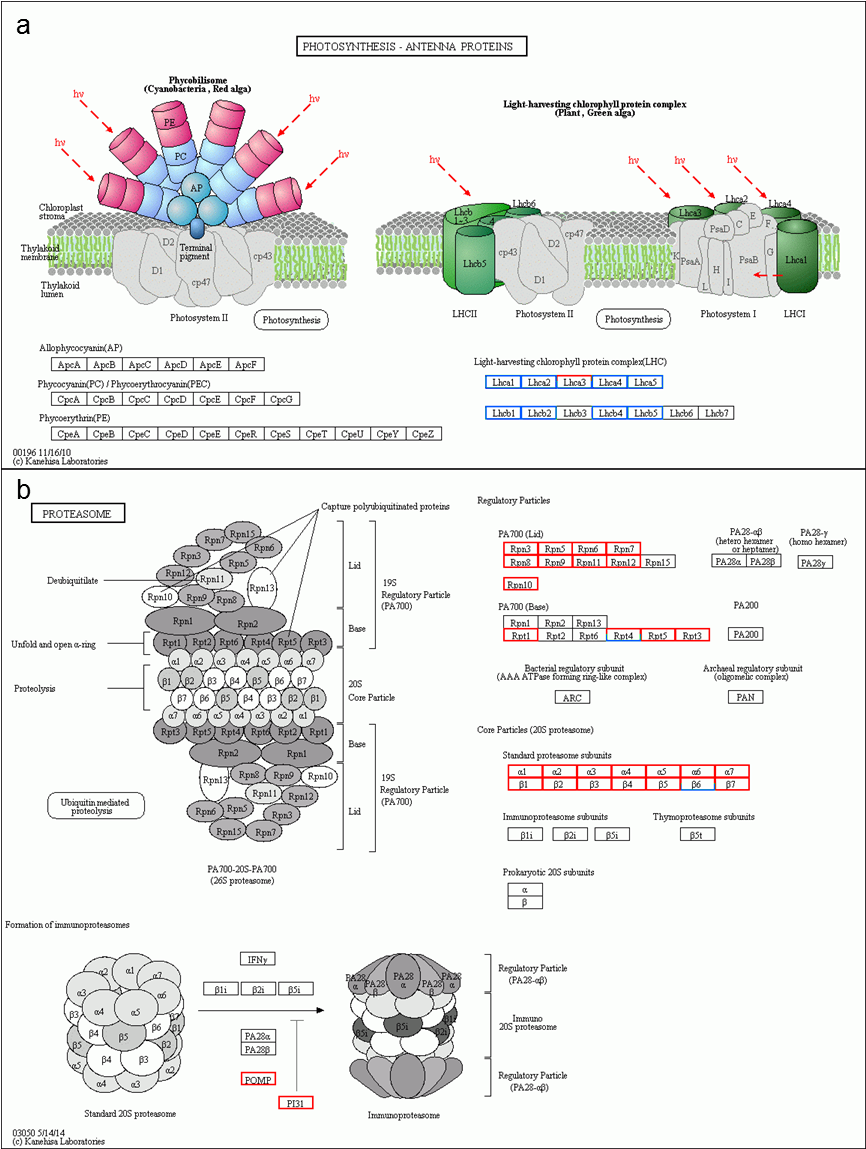


**Fig. S4. Transcriptional regulation at SS-6 h (0 h as control). (a) KEGG pathway of “photosynthesis-antenna protein” (b) KEGG pathway of “proteasome”. The red and blue boxes indicate up-regulation and down-regulation respectively.**

**
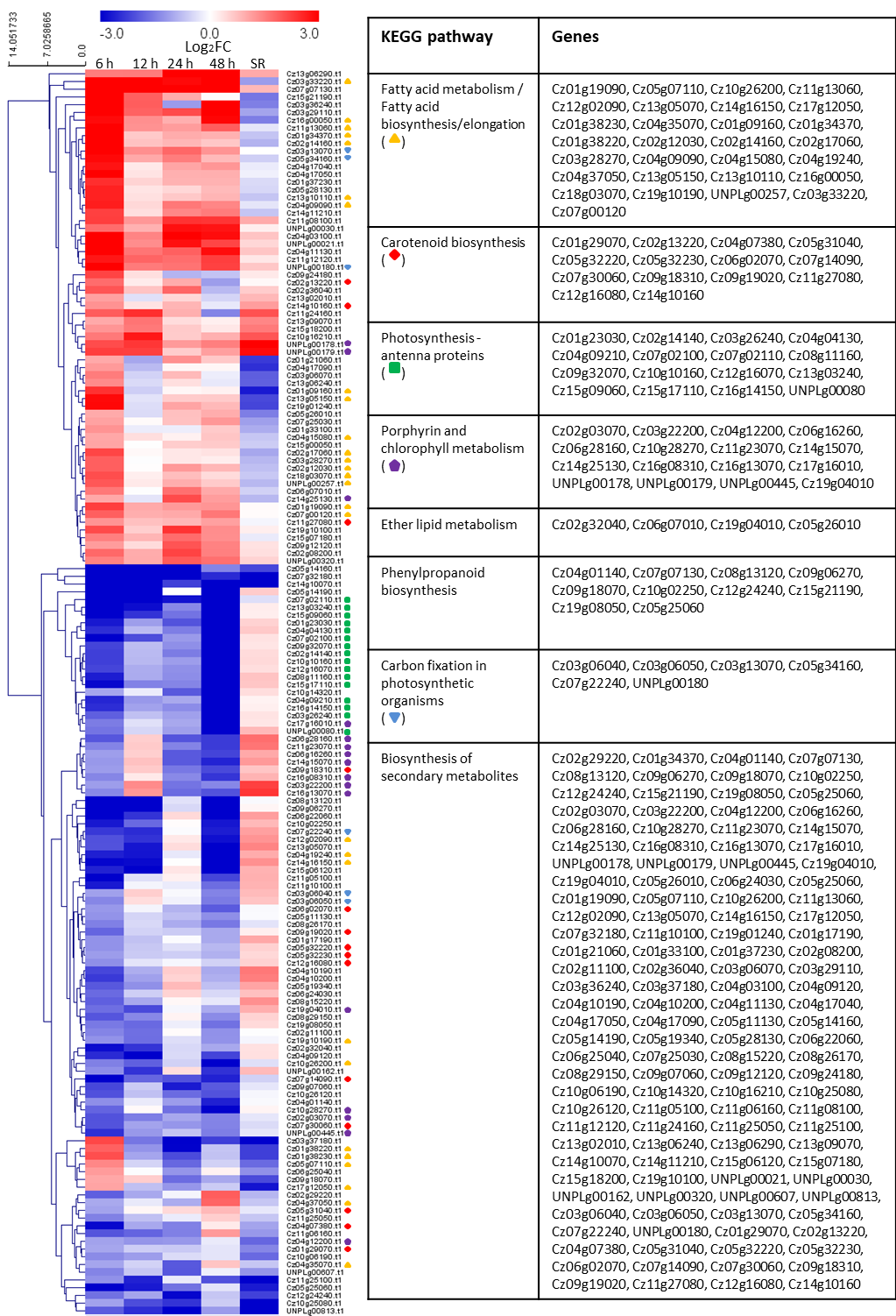
**

**Fig. S5. Hierarchical cluster analysis of DEGs responding to both SS and SR in enriched KEGG pathways.**


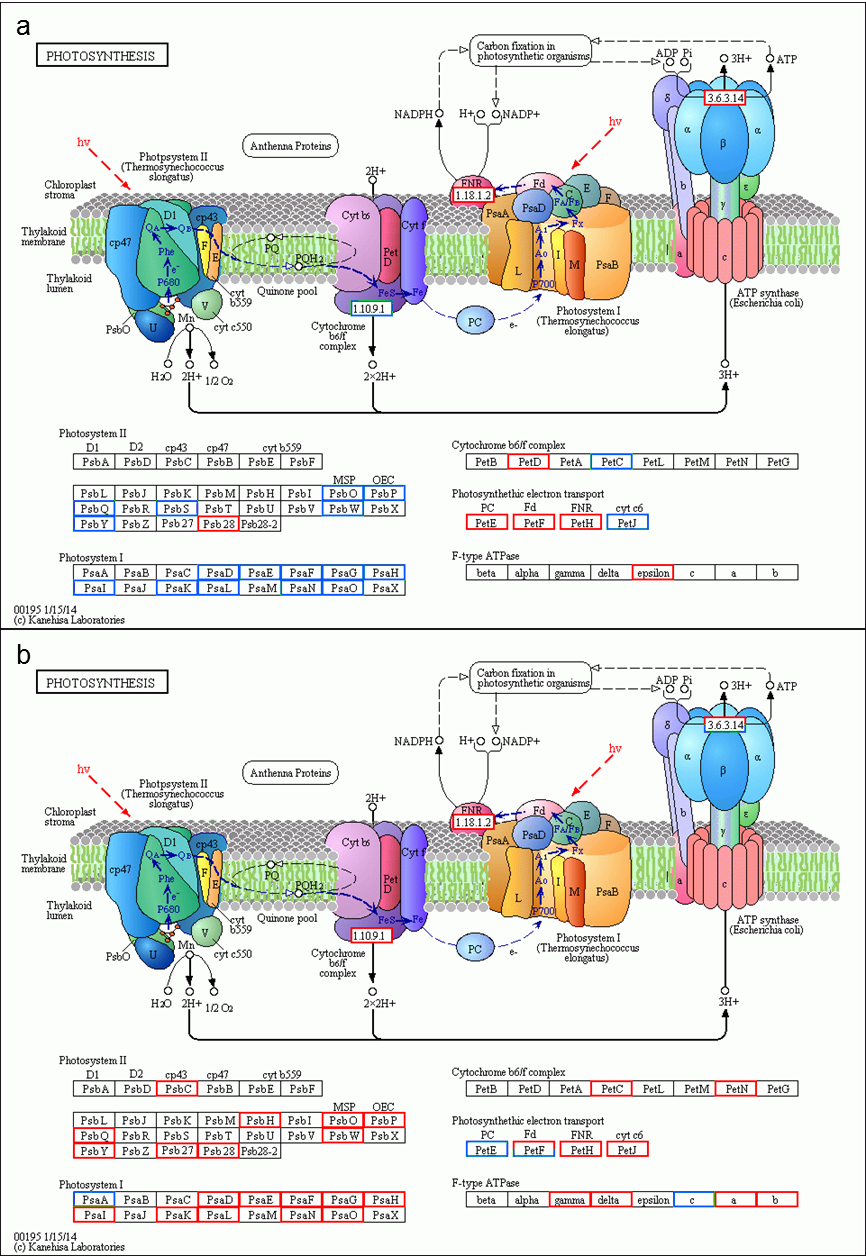


**Fig. S6. Transcriptional regulation in KEGG pathway of “photosynthesis”. (a) SS-6 h (0 h as control) (b) SR-12 h (SS-48 h as control). The red and blue boxes indicate up-regulation and down-regulation respectively.**

**
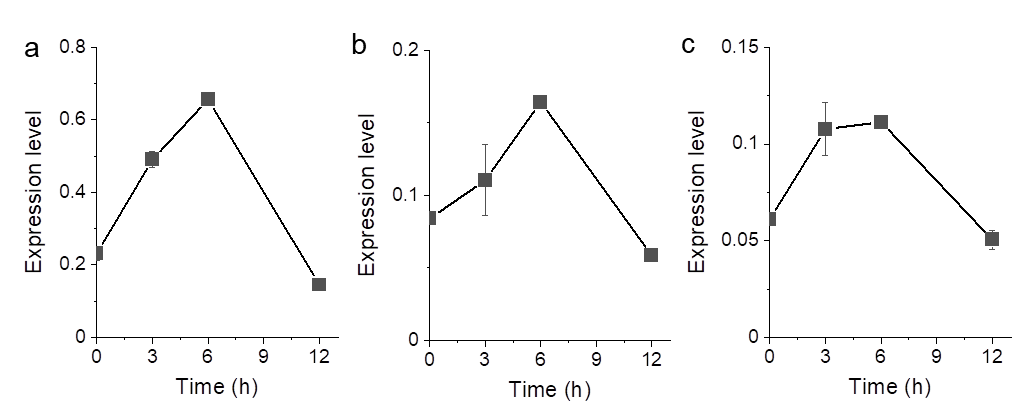
**

**Fig. S7. RT-PCR results of *β-CT* (a), *DGTT5* (b) and *BKT1* (c) at early SS.**

**Tables**

Table S1 Reads number and mapping ratio of RNA profiling

| Sample | Total Clean Reads (bp) |  | Genome Mapping | |  | Gene Mapping | | |
| --- | --- | --- | --- | --- | --- | --- | --- | --- |
|  |  |  | Total Mapping Ratio（%） | Uniquely Mapping Ratio（%） |  | Total Mapping Ratio（%） | Uniquely Mapping Ratio（%） | Gene number |
| 0 h-1 | 23960080 |  | 97.98 | 92.76 |  | 56.31 | 53.16 | 14682 |
| 0 h-2 | 23636340 |  | 97.07 | 91.07 |  | 56.03 | 52.70 | 14697 |
| 0 h-3 | 23677786 |  | 96.88 | 90.97 |  | 54.15 | 50.83 | 14684 |
| SS-6 h-1 | 23764698 |  | 96.38 | 90.77 |  | 42.54 | 40.62 | 14351 |
| SS-6 h-2 | 23805122 |  | 97.56 | 92.42 |  | 52.15 | 50.04 | 14457 |
| SS-6 h-3 | 23724876 |  | 97.22 | 91.85 |  | 53.48 | 51.4 | 14467 |
| SS-12 h-1 | 23591275 |  | 96.29 | 91.25 |  | 53.47 | 51.14 | 14521 |
| SS-12 h-2 | 23678281 |  | 96.63 | 91.50 |  | 54.76 | 52.43 | 14551 |
| SS-12 h-3 | 23623257 |  | 97.23 | 91.87 |  | 53.8 | 51.46 | 14541 |
| SS-24 h-1 | 23720077 |  | 97.11 | 91.88 |  | 55.88 | 53.49 | 14660 |
| SS-24 h-2 | 23759111 |  | 97.43 | 92.25 |  | 56.71 | 54.23 | 14647 |
| SS-24 h-3 | 23754453 |  | 96.99 | 91.72 |  | 56.64 | 54.24 | 14636 |
| SS-48 h-1 | 23688058 |  | 97.07 | 91.98 |  | 55.88 | 53.67 | 14867 |
| SS-48 h-2 | 23781211 |  | 97.21 | 91.87 |  | 56.85 | 54.59 | 14842 |
| SS-48 h-3 | 23345193 |  | 97.60 | 92.61 |  | 58.57 | 56.23 | 14802 |
| SR-12 h-1 | 23623462 |  | 96.78 | 90.84 |  | 56.83 | 53.57 | 14671 |
| SR-12 h-2 | 23686413 |  | 97.28 | 91.58 |  | 56.45 | 53.09 | 14653 |
| SR-12 h-3 | 23713087 |  | 97.34 | 91.69 |  | 57.29 | 53.91 | 14619 |

Total Clean Reads (bp): The reads amount after filtering, Unit: bp

Total Mapping Ratio: The percentage of mapped reads

Uniquely Mapping Ratio: The percentage of reads that map to only one location of reference

Table S2 Expression patterns of genes involved in TAG, astaxanthin biosynthesis and central carbon mechanism

| Gene ID | Annotation | Abbreviation | log 2 FC | | | | |
| --- | --- | --- | --- | --- | --- | --- | --- |
|  |  |  | SS-6 h | SS-12 h | SS-24 h | SS-48 h | SR-12 h |
| **FA biosynthesis** |  |  |  |  |  |  |  |
| Cz02g12030 | carboxyltransferase subunit alpha (ACCase complex) | *α-CT* | 1.89 | 0.03 | 1.21 | 0.86 | -0.75 |
| Cz02g17060 | carboxyltransferase subunit beta (ACCase complex) | *β-CT* | 2.20 | 0.09 | 0.29 | 0.35 | -1.03 |
| Cz03g28270 | biotin carboxyl carrier protein (ACCase complex) | *BCCP1* | 1.89 | -0.02 | 0.29 | 0.22 | -0.84 |
| Cz06g20040 | biotin carboxyl carrier protein (ACCase complex) | *BCCP2* | 2.76 | 0.50 | 0.55 | 0.76 | -1.14 |
| Cz13g10110 | biotin carboxylase (ACCase complex) | *BC* | 2.62 | 0.37 | 0.65 | 0.87 | -0.67 |
| Cz19g10190 | acetyl-CoA carboxylase | *ACCase* | -1.27 | -1.70 | -0.38 | -1.28 | -0.11 |
| Cz09g30220 | acyl carrier protein | *ACP1* | 2.98 | 1.08 | 0.60 | 0.54 | 0.20 |
| Cz07g17120 | acyl carrier protein | *ACP2* | 1.18 | 1.23 | 0.97 | 0.97 | 0.67 |
| Cz13g05150 | malonyl-CoA:Acyl carrier protein transacylase | *MCT1* | 2.90 | -0.33 | 0.62 | 0.63 | -2.13 |
| Cz04g37050 | malonyl-CoA:Acyl carrier protein transacylase | *MCT2* | -1.00 | -0.25 | -0.97 | 1.83 | -0.67 |
| UNPLg00257 | 3-oxoacyl-[acyl-carrier-protein] synthase, II (C16:0 to C18:0) | *KAS II* | 1.98 | 0.26 | 0.97 | 0.63 | -0.83 |
| Cz02g14160 | 3-oxoacyl-[acyl-carrier-protein] synthase, I (C4:0 to up to C16:0) | *KAS I* | 3.80 | 0.73 | 0.87 | 0.66 | -0.89 |
| Cz18g03070 | 3-oxoacyl-[acyl-carrier-protein] synthase, III (C2:0 to C4:0) | *KAS III* | 2.10 | 0.21 | 0.78 | 0.43 | -0.64 |

Table S2 (Continued) Expression patterns of genes involved in TAG, astaxanthin biosynthesis and central carbon mechanism

| Gene ID | Annotation | Abbreviation | log 2 FC | | | | |
| --- | --- | --- | --- | --- | --- | --- | --- |
|  |  |  | SS-6 h | SS-12 h | SS-24 h | SS-48 h | SR-12 h |
| Cz06g14030 | 3-oxoacyl-[acyl-carrier-protein] synthase, II | *KAS II* | 0.94 | 0.31 | 0.73 | 0.24 | 0.02 |
| Cz01g34370 | 3-oxoacyl-[acyl-carrier protein] reductase | *KAR1* | 3.45 | 0.64 | 0.98 | 0.93 | -0.69 |
| Cz16g00050 | 3-oxoacyl-[acyl-carrier protein] reductase | *KAR2* | 2.89 | 1.33 | 0.95 | 2.95 | -1.51 |
| Cz10g18200 | 3-oxoacyl-[acyl-carrier protein] reductase | *KAR3* | 1.19 | 1.38 | 0.09 | 0.06 | 0.34 |
| Cz03g39140 | 3-oxoacyl-[acyl-carrier protein] reductase? | *KAR4* | -0.16 | 0.59 | 0.68 | -0.27 | 1.09 |
| Cz15g00050 | 3-oxoacyl-[acyl-carrier protein] reductase | *KAR5* | 1.06 | 0 | 0.59 | 0.63 | -0.59 |
| Cz04g17270 | 3-oxoacyl-[acyl-carrier protein] reductase | *KAR6* | 0.50 | 0.44 | -0.04 | -0.37 | -0.26 |
| Cz11g27250 | 3-oxoacyl-[acyl-carrier protein] reductase? | *KAR7* | 0.35 | 0.57 | 0.14 | 1.24 | 0.06 |
| Cz01g09160 | 3-hydroxyacyl-[acyl-carrier-protein] dehydratase | *HAD* | 2.43 | -0.57 | 0.11 | 0.17 | -2.70 |
| Cz11g20040 | enoyl-[acyl-carrier protein] reductase | *ENR* | 2.14 | 0.44 | 0 | 0.23 | -0.61 |
| **FA desaturation** |  |  |  |  |  |  |  |
| Cz04g09090 | acyl-[acyl-carrier-protein] desaturase | *SAD1* | 2.67 | 0.46 | 1.60 | 1.43 | -0.31 |
| Cz13g17200 | acyl-[acyl-carrier-protein] desaturase | *SAD2* | -0.02 | -0.18 | 0.44 | 0.48 | 0.12 |
| Cz11g21120 | omega-6 fatty acid desaturase like | *FAD6 like* | 0.57 | 0.82 | 0.55 | 0.92 | 0.75 |

Table S2 (Continued) Expression patterns of genes involved in TAG, astaxanthin biosynthesis and central carbon mechanism

| Gene ID | Annotation | Abbreviation | log 2 FC | | | | |
| --- | --- | --- | --- | --- | --- | --- | --- |
|  |  |  | SS-6 h | SS-12 h | SS-24 h | SS-48 h | SR-12 h |
| Cz08g04110 | omega-6 fatty acid desaturase | *FAD6* | 1.01 | 0.53 | 0.04 | -0.31 | -0.55 |
| Cz03g33220 | omega-6 fatty acid desaturase | *FAD2* | 5.97 | 3.22 | 2.90 | 3.75 | -1.16 |
| Cz07g00120 | MGDG delta-7 desaturase | *FAD5* | 1.89 | 0.96 | 1.02 | 1.61 | 0.06 |
| Cz06g00170 | MGDG delta-7 desaturase | *FAD5* | -0.66 | -1.66 | -1.08 | -2.48 | -2.39 |
| Cz13g01140 | MGDG delta-7 desaturase like | *FAD5 like* | -1.56 | -0.53 | -1.18 | -0.56 | -0.68 |
| Cz06g12050 | MGDG delta-4 desaturase like | *delta4FAD* | -1.29 | -0.32 | -0.69 | -0.41 | 0.05 |
| UNPLg00012 | MGDG delta-4 desaturase like | *delta4FAD* | -1.07 | -0.30 | 0.67 | -0.46 | 2.09 |
| Cz12g10230 | delta-3 palmitate desaturase | *FAD4* | -1.31 | -0.89 | -1.22 | -1.08 | -0.30 |
| **TAG synthesis** |  |  |  |  |  |  |  |
| Cz11g03260 | glycerol-3-phosphate acyltransferase | *GPAT1* | -0.51 | 0.45 | -0.44 | 0.44 | 0.69 |
| Cz09g31330 | glycerol-3-phosphate acyltransferase | *GPAT2* | 0.73 | -0.25 | 0.34 | -0.15 | -0.39 |
| Cz16g02090 | 1-acyl-sn-glycerol-3-phosphate acyltransferase | *LPAAT1* | 0.61 | 0.15 | 0.34 | -0.04 | -0.04 |
| Cz10g20070 | 1-acyl-sn-glycerol-3-phosphate acyltransferase | *LPAAT2* | -0.18 | -0.8 | -0.25 | -0.48 | -0.49 |
| Cz05g23060 | phosphatidate phosphatase, Lipin | *PAP1* | -0.09 | -1.04 | 0.15 | -0.17 | -0.78 |

Table S2 (Continued) Expression patterns of genes involved in TAG, astaxanthin biosynthesis and central carbon mechanism

| Gene ID | Annotation | Abbreviation | log 2 FC | | | | |
| --- | --- | --- | --- | --- | --- | --- | --- |
|  |  |  | SS-6 h | SS-12 h | SS-24 h | SS-48 h | SR-12 h |
| Cz10g16040 | phosphatidate phosphatase | *PAP2* | 0.54 | 0.22 | -1.24 | -0.41 | -0.21 |
| Cz16g11240 | phosphatidate phosphatase | *PAP3* | 2.89 | 1.12 | 2.43 | 0.11 | 0.11 |
| Cz10g01120 | 2-acylglycerol O-acyltransferase | *MGAT* | -0.86 | -0.92 | -0.93 | -0.48 | -1.42 |
| Cz06g05010 | Diacylglycerol O-acyltransferase, type I | *DGAT1A* | -0.11 | -0.34 | 1.07 | 0.13 | 0.09 |
| Cz09g08290 | Diacylglycerol O-acyltransferase, type I | *DGAT1B* | -0.13 | -0.68 | -0.22 | -0.08 | -1.25 |
| Cz06g35060 | Diacylglycerol O-acyltransferase, type II | *DGTT1* | -0.52 | -1.62 | 0.24 | 0.28 | -2.21 |
| Cz06g22030 | Diacylglycerol O-acyltransferase, type II | *DGTT2* | -0.05 | 0.24 | -0.20 | 0.11 | -0.05 |
| Cz09g23010 | Diacylglycerol O-acyltransferase, type II | *DGTT3* | -0.93 | -0.77 | -0.11 | -1.22 | -0.33 |
| Cz11g24150 | Diacylglycerol O-acyltransferase, type II | *DGTT4* | -0.89 | -0.36 | -0.54 | -0.31 | -0.25 |
| Cz09g27290 | Diacylglycerol O-acyltransferase, type II | *DGTT5* | 1.75 | 0.11 | 1.03 | -0.03 | 0.68 |
| Cz15g22140 | Diacylglycerol O-acyltransferase, type II | *DGTT6* | -0.25 | -2.04 | 0.81 | -0.26 | 0.65 |
| Cz11g21100 | Diacylglycerol O-acyltransferase, type II | *DGTT7* | 1.81 | 0.17 | 0.81 | 0.19 | -0.33 |
| Cz08g14220 | Diacylglycerol O-acyltransferase, type II | *DGTT8* | 1.08 | -0.35 | 0.43 | -0.30 | -0.98 |
| Cz10g07180 | phospholipid:diacylglycerol acyltransferase | *PDAT* | 0.65 | -0.11 | 0.20 | -0.09 | -0.11 |

Table S2 (Continued) Expression patterns of genes involved in TAG, astaxanthin biosynthesis and central carbon mechanism

| Gene ID | Annotation | Abbreviation | log 2 FC | | | | |
| --- | --- | --- | --- | --- | --- | --- | --- |
|  |  |  | SS-6 h | SS-12 h | SS-24 h | SS-48 h | SR-12 h |
| Cz04g29220 | major lipid droplet protein | *MLDP* | 2.89 | 1.18 | 1.64 | 1.77 | -0.74 |
| **Carotenoids synthesis** | |  |  |  |  |  |  |
| Cz02g35280 | 1-deoxy-D-xylulose 5-phosphate synthase (DOXP synthase) | *DXS* | -0.18 | -0.05 | -0.82 | -1.43 | 0.08 |
| Cz07g19130 | 1-deoxy-D-xylulose 5-phosphate reductoisomerase | *DXR* | 0.36 | 0.76 | -0.31 | -0.17 | 0.60 |
| Cz12g10090 | 2-C-methyl-D-erythritol 4-phosphate cytidylyltransferase | *CMS* | -0.27 | 0.45 | -0.33 | 0.00 | 0.44 |
| Cz11g24270 | 4-diphosphocytidyl-2-C-methyl-D-erythritol kinase | *CMK* | -0.08 | 0.25 | -1.12 | -0.60 | -0.04 |
| Cz02g10180 | 2-C-methyl-D-erythritol 2,4-cyclodiphosphate synthase | *MCS* | 1.44 | 0.86 | 0.01 | 0.16 | 0.38 |
| Cz13g08020 | 4-hydroxy-3-methylbut-2-en-1-yl diphosphate synthase (HMB-PP synthase, IspG) | *HDS* | 0.81 | 0.40 | 0.09 | 0.17 | 0.15 |
| Cz05g23010 | 4-hydroxy-3-methylbut-2-enyl diphosphate reductase | *HDR* | 0.36 | 0.26 | 0.38 | 0.33 | 0.06 |
| Cz04g25080 | Acetoacetyl-CoA thiolase | *AACT* | 0.32 | 0.16 | 0.79 | 0.53 | -0.48 |
| Cz19g02060 | Hydroxymethylglutaryl-CoA synthase (HMG-CoA synthase) | *HCS* | -0.20 | -0.23 | 0.42 | -0.05 | -0.28 |
| Cz03g02150 | Isopentenyl-diphosphate Delta-isomerase | *IPPI* | 1.26 | 0.63 | 0.36 | 0.40 | 0.19 |

Table S2 (Continued) Expression patterns of genes involved in TAG, astaxanthin biosynthesis and central carbon mechanism

| Gene ID | Annotation | Abbreviation | log 2 FC | | | | |
| --- | --- | --- | --- | --- | --- | --- | --- |
|  |  |  | SS-6 h | SS-12 h | SS-24 h | SS-48 h | SR-12 h |
| Cz08g23110 | Isopentenyl-diphosphate Delta-isomerase | *IPPI1* | 1.35 | 0.16 | 0.54 | 0.15 | -0.74 |
| Cz03g10110 | Isopentenyl-diphosphate Delta-isomerase (IPP and DMAPP interconversion) | *IPPI2* | 0.09 | -0.24 | 0.72 | 0.48 | -0.77 |
| Cz02g31110 | geranyl diphosphate synthase | *GPPS* | 0.44 | 0.25 | 0.12 | 0.12 | -0.14 |
| Cz01g03190 | farnesyl diphosphate synthase | *FPPS* | 1.45 | 0.65 | 0.61 | 0.61 | 0.24 |
| Cz02g19200 | geranylgeranyl diphosphate synthase | *GGPPS* | -0.15 | 0.48 | -0.47 | -0.26 | 0.65 |
| Cz05g32220 | phytoene synthase (chloroplastic) | *PSY* | -1.28 | -0.65 | -0.37 | -0.83 | 0.54 |
| Cz02g32280 | phytoene desaturase | *PDS* | -1.38 | -0.16 | -1.50 | -0.22 | 0.65 |
| Cz10g17010 | Zeta-carotene desaturase (chloroplastic) | *ZDS* | 0.75 | 0.65 | 0.05 | 0.31 | 0.37 |
| Cz10g17130 | Zeta-carotene isomerase | *ZISO* | 0.86 | 1.07 | -0.20 | 0.26 | 0.42 |
| Cz16g01210 | carotenoid isomerase | *CRTISO1* | 0.15 | 0.53 | -0.06 | 0.53 | 0.35 |
| Cz12g03260 | carotenoid isomerase | *CRTISO2* | 0.03 | 0.24 | -0.31 | 0.11 | -0.30 |
| Cz14g22040 | carotenoid isomerase | *CRTISO3* | 2.06 | 0.67 | -0.06 | 0.13 | 0.07 |
| Cz09g18310 | Lycopene epsilon cyclase | *LCYe* | -1.16 | 0.63 | -1.59 | -2.14 | 0.68 |

Table S2 (Continued) Expression patterns of genes involved in TAG, astaxanthin biosynthesis and central carbon mechanism

| Gene ID | Annotation | Abbreviation | log 2 FC | | | | |
| --- | --- | --- | --- | --- | --- | --- | --- |
|  |  |  | SS-6 h | SS-12 h | SS-24 h | SS-48 h | SR-12 h |
| Cz12g10170 | lycopene beta cyclase | *LCYb* | 1.10 | 0.18 | 0.66 | 0.74 | 0.10 |
| Cz13g16110 | cytochrome P450 beta hydroxylase,CYP97A5(Cr) | *CYP97A* | 0.30 | 1.01 | -0.92 | 0 | 0.90 |
| Cz09g07100 | cytochrome P450 epsilon hydroxylase, CYP97C3 | *CYP97C* | -0.36 | 0.41 | -0.93 | 0 | 0.21 |
| Cz09g14130 | Cytochrome P450 CYP97B, beta or seta-rings (At) | *CYP97A* | -0.59 | 0.30 | -0.53 | -0.40 | 0.61 |
| Cz12g16080 | beta-carotene hydroxylase | *CHYb* | -1.31 | -0.32 | -0.10 | -0.54 | 0.55 |
| Cz13g13100 | beta-carotene ketolase/oxygenase | *BKT1* | 1.29 | 0.45 | 0.66 | -0.82 | 0.45 |
| Cz04g11250 | beta-carotene ketolase/oxygenase | *BKT2* | -0.49 | -1.00 | -0.19 | -1.77 | -0.73 |
| Cz07g30060 | zeaxanthin epoxidase | *ZEP* | -2.02 | -1.07 | -1.23 | -1.45 | -0.42 |
| Cz06g02070 | violaxanthin de-epoxidase | *VDE* | -1.21 | -0.13 | -0.89 | -1.94 | -0.07 |
| Cz15g04070 | neoxanthin synthase | *NXS* | -0.11 | 0.16 | 0.23 | -0.31 | 0.06 |
| Cz02g29020 | Long-chain-alcohol O-fatty-acyltransferase | *AAT?* | -0.34 | -0.58 | 0.59 | -0.12 | -0.76 |
| UNPLg00514 | Carotenoid cleavage dioxygenase | *CCD* | -0.23 | -0.35 | -0.42 | 0.03 | -0.46 |
| Cz04g35010 | Carotenoid cleavage dioxygenase | *CCD* | 0.38 | -0.28 | 0 | 0.72 | -0.20 |
| **OPP Pathway** |  |  |  |  |  |  |  |

Table S2 (Continued) Expression patterns of genes involved in TAG, astaxanthin biosynthesis and central carbon mechanism

| Gene ID | Annotation | Abbreviation | log 2 FC | | | | |
| --- | --- | --- | --- | --- | --- | --- | --- |
|  |  |  | SS-6 h | SS-12 h | SS-24 h | SS-48 h | SR-12 h |
| Cz06g12080 | Glucose-6-phosphate 1-dehydrogenase | *G6PD1* | 0.40 | -0.23 | 0.25 | -0.23 | -0.88 |
| Cz03g12030 | Glucose-6-phosphate 1-dehydrogenase | *G6PD2* | 1.31 | 0.47 | -0.27 | 0.17 | -0.16 |
| Cz04g18010 | 6-phosphogluconolactonase | *PGLS1* | 1.21 | 0.81 | 0.19 | 0.63 | 0.24 |
| Cz11g03250 | 6-phosphogluconolactonase | *PGLS2* | 1.90 | 0.25 | 0.95 | -0.63 | 0.46 |
| Cz05g06160 | 6-phosphogluconate dehydrogenase | *6PGD* | 2.23 | 0.71 | 0.75 | 0.61 | -0.07 |
| Cz05g13260 | ribose 5-phosphate isomerase | *RPI1* | -0.90 | 0.20 | 0.59 | 1.12 | -0.65 |
| Cz09g17220 | ribose 5-phosphate isomerase | *RPI2* | -0.06 | 0.67 | -0.07 | -0.57 | 0.69 |
| Cz01g08190 | ribose 5-phosphate isomerase | *RPI3* | 0.27 | 0.77 | -0.51 | 0.12 | 0.28 |
| Cz05g11190 | ribulose-phosphate 3-epimerase | *RPE1* | 0.78 | 0.43 | -0.49 | -0.13 | -0.23 |
| Cz04g31230 | ribulose-phosphate 3-epimerase | *RPE2* | 1.27 | 0.96 | -0.31 | 0.83 | 0.54 |
| Cz14g07140 | ribulose-phosphate 3-epimerase | *RPE3* | -0.62 | -0.56 | -0.46 | -0.29 | -0.11 |
| Cz03g04080 | transketolase | *TRK* | 0.72 | 0.56 | -0.23 | -0.17 | 0.22 |
| Cz06g25040 | transaldolase | *TAL1* | 1.18 | -0.03 | -1.24 | 0.16 | -1.30 |
| Cz04g12210 | transaldolase | *TAL2* | 0.59 | 0.48 | 0.14 | -0.47 | -0.34 |

Table S2 (Continued) Expression patterns of genes involved in TAG, astaxanthin biosynthesis and central carbon mechanism

| Gene ID | Annotation | Abbreviation | log 2 FC | | | | |
| --- | --- | --- | --- | --- | --- | --- | --- |
|  |  |  | SS-6 h | SS-12 h | SS-24 h | SS-48 h | SR-12 h |
| Cz12g20290 | transaldolase | *TAL3* | 0.77 | -0.32 | 0.50 | -0.24 | -0.75 |
| **Glycolysis and Glucogenesis** | |  |  |  |  |  |  |
| Cz13g07170 | hexokinase | *HK* | 0.73 | -0.34 | 0.33 | -0.77 | -0.54 |
| Cz06g03010 | glucokinase | *GK* | -0.43 | 0.08 | 0.05 | -0.11 | 0.27 |
| Cz01g30020 | glucose-6-phosphate isomerase | *PGI* | 1.15 | 0.55 | 0.28 | -0.10 | 0.35 |
| Cz09g25120 | 6-phosphofructokinase | *PFK1* | 0.16 | -0.59 | 0.28 | -0.45 | -0.68 |
| Cz16g11260 | 6-phosphofructokinase | *PFK2* | -0.23 | -0.25 | 0.38 | 0.23 | -0.58 |
| Cz07g13120 | 6-phosphofructokinase | *PFK3* | 0.29 | -0.16 | 0.36 | 0.13 | -0.15 |
| Cz04g03070 | Fructose-1,6-bisphosphatase, class I | *FBP1* | -0.95 | 0.20 | -0.90 | -1.32 | 0.37 |
| Cz01g04250 | Fructose-1,6-bisphosphatase, class II | *FBP2* | -0.24 | 0.52 | 0.19 | -0.37 | 0.19 |
| Cz05g01180 | Fructose-1,6-bisphosphatase, class II | *FBP3* | -0.33 | -0.54 | -0.55 | -0.50 | -1.92 |
| Cz05g37140 | Fructose-bisphosphate aldolase | *FBA1* | 0.72 | 0.26 | -0.93 | -1.48 | -0.07 |
| Cz06g07090 | Fructose-bisphosphate aldolase | *FBA2* | -0.26 | 0.20 | -0.48 | -0.35 | 0.12 |
| Cz03g06050 | Fructose-bisphosphate aldolase | *FBA3* | -1.42 | 0.40 | -0.16 | -1.55 | 0.70 |

Table S2 (Continued) Expression patterns of genes involved in TAG, astaxanthin biosynthesis and central carbon mechanism

| Gene ID | Annotation | Abbreviation | log 2 FC | | | | |
| --- | --- | --- | --- | --- | --- | --- | --- |
|  |  |  | SS-6 h | SS-12 h | SS-24 h | SS-48 h | SR-12 h |
| Cz03g13070 | Fructose-bisphosphate aldolase | *FBA4* | 3.27 | 1.32 | 1.79 | 1.27 | 0.01 |
| Cz06g17270 | triosephosphate isomerase | *TIM* | 1.03 | 1.17 | 0.51 | 0.83 | 1.14 |
| Cz05g34160 | glyceraldehyde 3-phosphate dehydrogenase (NAD) | *GAPDH* | 2.91 | 1.01 | 1.54 | 0.81 | -0.56 |
| Cz16g19260 | phosphoglycerate kinase | *PGK* | -0.43 | 0.11 | -0.55 | -1.53 | 0.41 |
| Cz06g01110 | phosphoglycerate mutase, 2,3-bisphosphoglycerate-dependent | *PGAM1* | 3.54 | 1.21 | 1.18 | 0.96 | 0.01 |
| Cz09g03220 | phosphoglycerate mutase, 2,3-bisphosphoglycerate-independent | *PGAM2* | -0.60 | -0.35 | 0.10 | -0.28 | -0.34 |
| Cz04g11130 | phosphoglycerate mutase, 2,3-bisphosphoglycerate-independent | *PGAM3* | 4.01 | 1.63 | 1.88 | 2.86 | 0.69 |
| Cz08g21030 | phosphoglycerate mutase, 2,3-bisphosphoglycerate-independent | *PGAM4* | 0.86 | 0.26 | -0.20 | -0.19 | -0.22 |
| Cz14g01200 | phosphoglycerate mutase | *PGAM5* | 0.25 | 0.12 | -0.71 | -0.05 | -0.23 |
| Cz14g08090 | phosphoglycerate mutase | *PGAM6* | -0.18 | 0.13 | -0.21 | -0.23 | 0.00 |
| Cz01g38050 | phosphoglycerate mutase | *PGAM7* | 0.51 | -0.37 | 0.33 | 0.13 | -0.39 |

Table S2 (Continued) Expression patterns of genes involved in TAG, astaxanthin biosynthesis and central carbon mechanism

| Gene ID | Annotation | Abbreviation | log 2 FC | | | | |
| --- | --- | --- | --- | --- | --- | --- | --- |
|  |  |  | SS-6 h | SS-12 h | SS-24 h | SS-48 h | SR-12 h |
| Cz05g10010 | enolase | *ENO* | 1.19 | 0.35 | 0.75 | 0.62 | -0.31 |
| UNPLg00362 | pyruvate kinase | *PK1* | 0.27 | 0.07 | -0.48 | 0.02 | -0.39 |
| Cz08g12170 | pyruvate kinase | *PK2* | -0.48 | -0.16 | -0.42 | 0.15 | -0.86 |
| Cz16g00040 | pyruvate kinase | *PK3* | 2.85 | 1.38 | 1.16 | 1.42 | 0.82 |
| Cz14g14130 | pyruvate kinase | *PK4* | -0.22 | 0.08 | 0.23 | 0.40 | -0.20 |
| Cz04g17050 | pyruvate kinase | *PK5* | 3.02 | 0.34 | 0.93 | 0.95 | -0.17 |
| Cz01g21060 | pyruvate kinase | *PK6* | 1.02 | -1.04 | 1.31 | 0.66 | -2.31 |
| Cz15g09100 | pyruvate kinase | *PK7* | 1.70 | 0.58 | 0.06 | 0.80 | 0.17 |
| Cz10g06190 | pyruvate kinase | *PK8* | -1.35 | -1.14 | -0.42 | -0.31 | -1.76 |
| Cz01g05160 | phosphoenolpyruvate carboxykinase (ATP) | *PEPCK* | 0.04 | 0.48 | 0.59 | -0.10 | -0.84 |
| Cz04g02090 | pyruvate carboxylase | *PYC* | -0.90 | -0.25 | -0.34 | 0.22 | 0.20 |
| **TCA** |  |  |  |  |  |  |  |
| Cz02g27080 | citrate synthase | *CIS1* | 1.38 | 0.84 | 0.34 | 0.02 | 0.11 |
| Cz02g12210 | citrate synthase | *CIS2* | 1.66 | 0.92 | 1.46 | 0.74 | -0.05 |

Table S2 (Continued) Expression patterns of genes involved in TAG, astaxanthin biosynthesis and central carbon mechanism

| Gene ID | Annotation | Abbreviation | log 2 FC | | | | |
| --- | --- | --- | --- | --- | --- | --- | --- |
|  |  |  | SS-6 h | SS-12 h | SS-24 h | SS-48 h | SR-12 h |
| Cz13g00140 | aconitate hydratase | *ACH* | 0.31 | -0.01 | 0.11 | -0.01 | -0.43 |
| Cz11g28180 | isocitrate dehydrogenase, NAD-dependent | *IDH1* | 0.95 | 0.03 | 0.33 | 0.21 | -0.66 |
| Cz11g08120 | isocitrate dehydrogenase, NAD-dependent | *IDH2* | 0.66 | 0.22 | -0.06 | -0.24 | -0.30 |
| Cz12g16160 | isocitrate dehydrogenase, NADP-dependent | *IDH3* | 0.84 | 0.76 | 0.16 | 0.19 | 0.49 |
| Cz05g03220 | 2-oxoglutarate dehydrogenase, E1 | *OGDH1* | 1.37 | -0.07 | 0.31 | -0.20 | -0.63 |
| Cz01g28100 | 2-oxoglutarate dehydrogenase, E1 | *OGDH2* | -0.89 | -0.78 | -0.43 | -0.24 | -0.22 |
| Cz02g11010 | 2-oxoglutarate dehydrogenase, E2 | *OGDH3* | 0.87 | 0.39 | 0.06 | -0.29 | 0.11 |
| Cz01g33150 | succinyl-CoA synthetase, alpha subunit | *SCSa* | 0.32 | 0.36 | 0.15 | -0.07 | -0.35 |
| Cz03g31200 | succinyl-CoA synthetase, beta subunit | *SCSb* | 0.27 | -0.08 | 0.19 | -0.30 | -0.88 |
| Cz03g18320 | succinate dehydrogenase (ubiquinone) flavoprotein subunit | *SDH1* | 0.14 | 0.17 | 0.15 | -0.30 | -0.10 |
| Cz15g13230 | succinate dehydrogenase (ubiquinone) iron-sulfur subunit | *SDH2* | 1.00 | 0.26 | 0.57 | -0.34 | -0.07 |
| Cz07g14020 | succinate dehydrogenase (ubiquinone) cytochrome b560 subunit | *SDH3* | 1.22 | 0.57 | 0.80 | 0.31 | -0.20 |
| Cz07g14015 | succinate dehydrogenase (ubiquinone) membrane anchor subunit | *SDH4* | 0.95 | 0.79 | 0.50 | 0.04 | 0.02 |

Table S2 (Continued) Expression patterns of genes involved in TAG, astaxanthin biosynthesis and central carbon mechanism

| Gene ID | Annotation | Abbreviation | log 2 FC | | | | |
| --- | --- | --- | --- | --- | --- | --- | --- |
|  |  |  | SS-6 h | SS-12 h | SS-24 h | SS-48 h | SR-12 h |
| Cz07g04030 | fumarate hydratase, class I | *FHD* | 0.18 | 0.58 | 0.20 | 0.14 | 0.18 |
| Cz02g21340 | malate dehydrogenase (NAD) | *MDH* | 1.01 | 1.14 | 0.37 | 0.13 | 0.66 |
| **Acetyl CoA source** |  |  |  |  |  |  |  |
| Cz18g13050 | pyruvate dehydrogenase, E1 alpha | *PDHC1* | 0.32 | 0.37 | 0.19 | 0.00 | 0.01 |
| Cz07g25130 | pyruvate dehydrogenase, E1 beta | *PDHC2* | 0.64 | 0.57 | 0.35 | -0.14 | 0.16 |
| Cz03g08090 | pyruvate dehydrogenase, E1 alpha | *PDHC3* | 2.15 | 0.17 | 0.79 | 0.68 | -0.30 |
| Cz01g37230 | pyruvate dehydrogenase, E1 beta | *PDHC4* | 2.47 | 0.52 | 0.91 | 0.91 | -0.43 |
| Cz05g28130 | pyruvate dehydrogenase, E2 (dihydrolipoamide acetyltransferase) | *PDHC5* | 2.61 | 0.32 | 0.47 | 0.81 | -0.46 |
| Cz10g25060 | pyruvate dehydrogenase, E3 (dihydrolipoamide dehydrogenase) | *PDHC6* | 0.53 | 0.43 | 0.34 | -0.05 | 0.08 |
| Cz07g16120 | pyruvate dehydrogenase, E3 (dihydrolipoamide dehydrogenase) | *PDHC7* | 1.68 | -0.11 | 0.32 | 0.49 | -0.55 |
| Cz01g33100 | Pyruvate decarboxylase | *PDC* | 1.28 | -0.22 | -0.21 | 0.63 | -0.97 |

Table S2 (Continued) Expression patterns of genes involved in TAG, astaxanthin biosynthesis and central carbon mechanism

| Gene ID | Annotation | Abbreviation | log 2 FC | | | | |
| --- | --- | --- | --- | --- | --- | --- | --- |
|  |  |  | SS-6 h | SS-12 h | SS-24 h | SS-48 h | SR-12 h |
| Cz11g04290 | aldehyde dehydrogenase (NAD+) | *ALDH1* | -1.10 | 0.71 | 0.64 | 2.90 | 0.44 |
| Cz03g20090 | aldehyde dehydrogenase (NAD+) | *ALDH2* | -1.52 | -1.25 | -0.44 | -1.21 | -1.66 |
| Cz05g18170 | aldehyde dehydrogenase | *ALDH3* | 0.24 | 0.12 | -0.18 | -0.45 | -0.60 |
| Cz09g15060 | acetyl-CoA synthetase | *ACS1* | 0.81 | 0.42 | 0.60 | -0.03 | 0.02 |
| Cz12g10100 | acetyl-CoA synthetase | *ACS2* | 0.37 | -0.85 | -0.22 | -0.65 | -1.55 |

Table S3 Fatty acid composition of TAG and TFA after 4-days culture

| Fatty acids | TAG | | |  | TFA | | |
| --- | --- | --- | --- | --- | --- | --- | --- |
|  | Control (%) | SS (%) | SR (%) |  | Control (%) | SS (%) | SR (%) |
| C16:0 | 19.37±0.79 | 21.98±0.09 | 20.48±2.59 |  | 30.88±1.41 | 21.37±0.18 | 22.67±0.40 |
| C16:1 | 1.72±0.11 | 3.01±0.06 | 2.38±0.66 |  | 2.31±1.62 | 2.82±0.26 | 2.03±1.32 |
| C16:2 | 1.88±0.18 | 2.16±0.03 | 1.62±0.29 |  | 1.23±0.36 | 2.15±0.32 | 2.02±0.49 |
| C16:3 | 2.95±0.05 | 2.44±0.04 | 2.44±0.28 |  | 1.51±0.13 | 3.02±0.54 | 2.51±1.43 |
| C16:4 | 1.68±0.13 | 0.19±0.02 | 1.87±0.69 |  | 1.11±0.06 | 0.72±0.13 | 3.61±1.40 |
| C18:0 | 6.56±0.08 | 5.98±0.17 | 5.19±0.43 |  | 10.20±0.29 | 5.80±0.93 | 4.16±1.52 |
| C18:1 | 40.75±0.93 | 42.75±0.18 | 39.11±1.00 |  | 30.21±1.14 | 39.59±1.45 | 34.94±1.23 |
| C18:2 | 13.75±0.09 | 14.10±0.19 | 15.80±0.46 |  | 8.83±0.45 | 13.62±0.83 | 14.30±0.73 |
| C18:3 | 10.35±0.19 | 7.02±0.04 | 9.42±0.99 |  | 7.35±0.63 | 8.97±0.87 | 9.92±3.24 |
| C18:4 | 1.00±0.11 | 0.33±0.03 | 1.64±0.53 |  | 6.37±0.14 | 1.94±0.41 | 3.87±0.92 |

Table S4 ROS abundance in *C. zofingiensis* under different status

| Time (Days) | ROS abundance(Fluorescence Intensity/10^7 cells) | | |
| --- | --- | --- | --- |
| Phase 1 | Control | SS | |
| 0 | 716.6±18.9 | 702.3±23.3 | |
| 1 | 786.7±33.9 | 768.3±63.4 | |
| 2 | 873.0±29.1 | 1216.7±80.7 | |
| Phase 2 | Control | SS | SR |
| 3 | 669.7±36.2 | 1131.0±94.6 | 891.3±4.04 |
| 4 | 754.3±15.0 | 1246.6±20.2 | 634.7±20.2 |
